# Preclinical anticancer studies on the ethyl acetate leaf extracts of *Datura stramonium* and *Datura inoxia*

**DOI:** 10.1186/s12906-020-02975-8

**Published:** 2020-06-17

**Authors:** Bakht Nasir, Muhammad Waleed Baig, Muhammad Majid, Syeda Masooma Ali, Muhammad Zafar Irshad Khan, Syeda Tayyaba Batool Kazmi, Ihsan-ul Haq

**Affiliations:** 1 Department of Pharmacy, Faculty of Biological Sciences, Quaid-i-Azam University, Islamabad, 45320 Pakistan; 2 Department of Pharmacy, Capital University of Science and Technology, Islamabad, 44000 Pakistan

**Keywords:** *Datura stramonium*, *Datura inoxia*, Anticancer, Antileukemic, Breast cancer, Prostate cancer, Benzene toxicity

## Abstract

**Background:**

Cancer is a horrific disease relentlessly affecting human population round the globe. Genus *Datura* encompasses numerous species with reported medicinal uses. However, its potential as a source of natural anticancer agents is yet to be determined. *Datura stramonium* (DS) and *Datura inoxia* (DI) are the two species chosen for this study.

**Methods:**

Total phenolic and flavonoid content (TPC and TFC) as well as antioxidant activity were assessed through colorimetric method. Polyphenolic quantification was done by RP-HPLC. Following extract standardization ethyl acetate leaf extracts of both species (DSL-EA and DIL-EA) were chosen for anticancer studies. In vitro cytotoxicity using various models including cancer cell lines was monitored. Following toxicity studies, benzene (0.2 ml) was used to induce leukemia in Sprague-Dawley rats. Extracts were orally administered to preventive (100 and 200 mg/kg) and treatment (200 mg/kg only) groups. The antileukemic potential of extracts was assessed through haematological, biochemical, endogenous antioxidants and histological parameters.

**Results:**

Significant TPC and TFC were estimated in DSL-EA and DIL-EA. RP-HPLC quantified (μg/mg extract) rutin (0.89 ± 0.03), gallic acid (0.35 ± 0.07), catechin (0.24 ± 0.02) and apigenin (0.29 ± 0.09) in DSL-EA while rutin (0.036 ± 0.004) and caffeic acid (0.27 ± 0.03) in DIL-EA. Both extracts exhibited significant brine shrimp cytotoxicity (LC_50_ < 12.5 μg/ml). DIL-EA exhibited greater cytotoxicity against PC-3, MDA-MB 231 and MCF-7 cell lines (IC_50_ < 3 μg/ml in each case) as well as higher protein kinase inhibitory action (MIC: 25 μg/disc) compared to DSL-EA. Leukemia induced in rats was affirmed by elevated serum levels of WBCs (7.78 ± 0.012 (× 10^3^) /μl), bilirubin (7.56 ± 0.97 mg/dl), Thiobarbituric acid reactive substances (TBARs) (133.75 ± 2.61 nM/min/mg protein), decreased RBCs (4.33 ± 0.065 (× 10^6^)/μl), platelets (344 ± 3.19 (× 10^3^)/μl), total proteins (2.14 ± 0.11 g/dl), Glutathione S-transferases (GST) (81.01 ± 0.44 nM/min/ml), endogenous antioxidant enzymes levels and abnormal liver and kidney functionality in disease control rats. Both species revealed almost identical and significant (*p* < 0.05) alleviative effects in benzene induced leukemia.

**Conclusion:**

Comprehensive screening divulged the tremendous potential of selected species as potent source of natural anticancer agents in a variety of cancers particularly leukemia. Present study might provide useful finger prints in cancer research and mechanistic studies are prerequisite in logical hunt of this goal.

## Background

Cancer is undoubtedly one of the leading causes of death worldwide. Global cancer statistics show that in males lung, prostrate and colon cancer are more common while in females breast, stomach, colon, rectum and ovarian cancers predominate [1]. Out of the many prominent causes of cancer, chemical carcinogens, inactivation of tumor suppressor genes, chromosomal rearrangements, impulsive transformations and viruses have played central roles in the onset of the disease [2, 3]. Deviation from the normal process of DNA controlled cell division leads to cancer. Cell cycle process comprising of accurate DNA reproduction followed by production of ample organelles, cellular membranes and proteins for daughter cells and lastly, equal partition of cytoplasm and DNA during division must be monitored and looked after via a meticulous feedback control system. Any disorientation in the sequence of the aforementioned molecular steps leads to uncontrolled cell division and the onset of malignancy [3].

Normal cell is believed to transform into a cancerous cell after passing through numerous stages and the process may take several years or even decades to complete. Initiation, promotion and progression are the three important stages of carcinogenesis [4]. Averting initiation is an imperative strategy in anticancer therapy, likewise, curbing cancer in the stages following initiation. Oxidative damage to DNA is implicated as one of the major underlying mechanisms contributing to cancer. Division of cells carrying damaged DNA prior to its repair can lead to perpetual genetic alteration which is undoubtedly the initial step in carcinogenesis [3, 5].

The most commonly diagnosed and second leading cause of cancer related deaths in males is prostate cancer, while in females it is breast cancer. Notwithstanding the tireless efforts made to enhance their early diagnosis and treatment, an imperative need still exists for development of alternative therapeutic targets and new markers for effective management of both prostate and breast cancer patients [6, 7]. Women in both high and low resource settings are equally affected and it accounts for a major health burden worldwide [8]. It is a prime cause of death from cancers in women with over 1.1 million diagnosed cases and more than 410,000 deaths worldwide [9]. Furthermore, leukemia is a type of cancer that starts in the bone marrow, but in most cases quickly moves into the blood. The exact cause of leukemia is still unknown. Viral, genetic, environmental or immunological factors may be involved [10]. It is globally recognized as one of the deadliest cancer type in people of every age and ethnicity, so great efforts are made in pursuit of curative strategies against this deadly disease [11]. Abovementioned cancer types are thus targeted in current study using relevant in vitro and in vivo models.

Unfortunately, advancements in understating the molecular mechanism of cancer are not going to bring down the unacceptably higher mortality rates, unless and until the role of natural products in chemoprevention is revisited and revitalized. A large number of compounds used currently for cancer treatment and prevention are derived from natural sources including plants, animals and microorganisms. Plant based anticancer agents including, but not limited to vincristine, vinblastine, irinotecan, etoposide, paclitaxel etc. have served a great purpose in cancer therapeutics [3, 12].

Genus *Datura* (family Solanaceae) consists of all the nightshades and numerous agricultural plants. This genus contains medicinally important species, the most important ones being *D. stramonium, D. inoxia, D. metel, D. ferox, D. ceratocaula*. It has global geographical distribution and can be found throughout America, Europe and Asia either as native or adventive plants, moreover, some species have also been reported in Africa and Australia. *Datura* species have been used for their medicinal and recreational purposes since antiquity. Most common ethnopharmacological and traditional uses of these plants include; anesthetic, hypnotic, sedative, expectorant, demulcent, antihemorrhoidal, antiasthmatic and antitumor [13]. These plants are known predominantly for their alkaloidal content, most important of which are the tropane alkaloids namely hyoscyamine, hyoscine and atropine. *Datura* is also acclaimed for presence of withanolides, which constitute a large family of plant steroids and steroidal glycosides [14, 15].

*D. inoxia* Mill. and *D. stramonium* Linn. are the two species selected to carry out this research based on their ethnomedicinal properties and their incomplete appraisal as potential anticancer agents. Nonetheless, there is evidence to suggest their potential as sources of anticancer compounds. *D. inoxia* was found to be the most potent source of antioxidants when equated with other species of *Datura* in a comparative analysis of their free radical scavenging property towards 2, 2-diphenyl-1-picrylhydrazyl (DPPH) a stable free radical [16]. Anticancer effects were also studied against human colon adenocarcinoma and larynx cancer cell lines and satisfactory results were observed. Likewise, dinoxin B, a withanolide recently isolated from *D. inoxia,* demonstrated significant anticancer activity when tested against several human cancer cell lines [14]. There are few studies regarding the anticancer potential of *D. stramonium* and it has shown noteworthy in vitro antioxidant and anticancer activity against the tested cancer cell lines [17, 18].

This work describes our findings in relation to the potential usage of extracts of *D. inoxia* and *D. stramonium* against kinase inhibitory, cytotoxicity against *Artemia salina*, normal human lymphocytes, cancer cell lines and in vivo antileukemic action of the two designated species and correlates the observed activities to estimated phytochemicals and the already reported secondary metabolites. Antileukemic action was evaluated using benzene induced leukemia model in male Sprague Dawley rats in an effort to associate the in vitro findings with potential anticancer effects of selected samples in relevant in vivo model.

## Methods

### Plant collection and preparation of extract

*D. inoxia* was collected from Quaid-i-Azam University, Islamabad Capital Territory, Pakistan, while *D. stramonium* was collected from Oghi town in dictrict Mansehra, Khyber Pakhtunkhwa, Pakistan in august 2016. *D. stramonium* was collected from its wild natural habitat and is not an endangered species thus no permission was required from any state agency. The collected plants were identified by Prof. Dr. Rizwana Aleem Qureshi, Department of Plant Sciences, Faculty of Biological Sciences, Quaid-i-Azam University Islamabad, Pakistan. Dried sample of both plants were deposited at the Herbarium of Quaid-i-Azam University, Islamabad, with voucher numbers PHM-487 for *D. inoxia* and PHM-504 for *D. stramonium.* Collected plants were washed with water. Leaf, fruit, steam and root parts were separated and shade dried at ambient temperature with adequate ventilation until fully dried out. Drying was followed by grinding the segregated plant parts into fine powder. Successive extraction at room temperature by ultra-sonication aided maceration was performed using n-hexane, ethyl acetate, methanol and distilled water as solvents. The process of extraction was repeated twice with each solvent and extracts were filtered using Whatmann No.1 filter paper. Extracts were latter concentrated in a rotary evaporator and dried in vacuum oven at 40 °C. Samples of different concentrations were prepared and numerous bench top assays were performed in triplicate. Percent extract recovery (%w/w) was calculated using the following formula;

$$ \% Extrac\mathrm{t}\  recovery=\frac{A}{B}\times 100 $$

Where A = weight of dry extract and B = weight of powdered plant material.

### Chemicals and reagents

All chemicals and reagents used in current study were of analytical grade. Solvents i.e. n-hexane, ethyl acetate, methanol and dimethylsulfoxide (DMSO) were purchased from Merck (Darmstadt, Germany). Folin-Ciocalteu reagent and DPPH were purchased from Sigma–Aldrich (Steinheim, Germany). Potassium dihydrogen phosphate, dipotassium hydrogen phosphate, ferrous chloride, sodium hydroxide, aluminum chloride, ascorbic acid, quercetin, gallic acid, rutin, caffeic acid, kaempferol, myricetin and (+)-catechin were acquired from Sigma–Aldrich (Steinheim, Germany). Tween 80, thiobarbituric acid, tricholoroacetic acid, ferric chloride and phenazine methosulphate were acquired from Sigma (Chemicals Co. St. Louis, USA). Sodium carbonate, sulphuric acid, hydrogen peroxide, potassium ferricyanide, sodium dihydrogen phosphate and disodium hydrogen phosphate were purchased from Merck KGaA (Darmstadt, Germany).

### Animals

Brine shrimp eggs were acquired from Oceans Star International USA (O.S.I®). Sprague Dawley rats were purchased from National Institute of Health, Islamabad, Pakistan (NIH).

### Phytochemical analysis

#### Total phenolic content estimation (TPC)

Standard procedure previously reported by Humaira et al., [19] was followed using Folin–Ciocalteu reagent for the determination of TPC. A stock solution (4 mg/ml) of each test sample was prepared in DMSO and an aliquot of 20 μl from the stock solution was transferred to respective wells of a 96 well plate. It was followed by addition of 90 μl of Folin–Ciocalteu reagent. After incubation at room temperature for 5 min, 90 μl of sodium carbonate was added to wells carrying the reaction mixture. Absorbance was measured at 630 nm using microplate reader (Elx 800, Biotech USA). The experiment was run in triplicate and a calibration curve (y = 0.0567x – 0.0405, R^2^ = 0.9927) was drawn using gallic acid (2.5, 5, 10, 20, 40 μg/ml) as positive control under identical operating conditions. Estimated TPC was expressed as μg gallic acid equivalent per mg extract (μg GAE/mg extract).

#### Total flavonoid content estimation (TFC)

Aluminum chloride base calorimetric method was used for the estimation of TFC [20]. The assay was executed in 96 well plate and 20 μl of test samples (4 mg/ml DMSO) were transferred to the wells. It was followed by addition of 10 μl each of 10% (w/v) aluminum chloride, 1.0 M potassium acetate and 160 μl of distilled water. Following incubation for 30 min at room temperature, absorbance of reaction mixture was taken at 415 nm using microplate reader. The experiment was run in triplicate and calibration curve (y = 0.0389x – 0.0187, R^2^ = 0.9948) was drawn using quercetin as positive control (2.5, 5, 10, 20, 40 μg/ml). Ensuing TFC was expressed as μg quercetin equivalent per mg extract (μg QE/mg extract).

#### High performance liquid chromatography (HPLC) analysis

The detection and quantification of polyphenols was performed by HPLC analysis of selected *Datura* species. Previously reported procedure by Bakht et al., [20] was followed with slight modifications as per system suitability. HPLC system, Agilent Chem station Rev. B.02–01-SR1 (260) was equipped with a Zorbex-C8 analytical column (4.6 × 250 nm, 5 μm particle size) in combination with a diode array detector (DAD; Agilent technologies, Germany). RP-HPLC analysis was carried out by using two mobile phases (A: acetonitrile:methanol:water:acetic acid in ratio 5:10:85:1 and B: acetonitrile:methanol:acetic acid in ratio 40:60:1) with a flow rate maintained at 1 ml/min. The gradient (A: B) employed was: 0–20 min (0–50% B), 20–25 min (50–100% B) and from 25 to 30 min it was 100% B. Volume of sample (10 mg/ml extracts) injected via an injection port into the column was 20 μl. Samples were filtered through a 0.45 μm membrane filter prior to injection and each sample run was followed by a 10 min column reconditioning step. Wavelengths used to detect the standards were 257 nm for Rutin, 279 nm for gallic acid and catechin, 325 nm for caffeic acid and apigenin, while quercetin, myricetin and kaempferol were analyzed at 368 nm. Identification and quantification of different polyphenols was accomplished by comparing the UV absorption spectra and retention time of samples with those of standards and the results were expressed as μg/mg extract.

### Estimation of in vitro antioxidant potential

#### Determination of total antioxidant capacity (TAC)

Total antioxidant capacity of extracts was determined by phosphomolybdenum based method described previously [21]. The assay was performed by mixing 100 μl of extracts (4 mg/ml DMSO) with a reagent solution consisting of 28 mM sodium phosphate, 4 mM ammonium molybdate and 0.6 M sulphuric acid. Reaction mixture was then incubated at 95 °C for 90 min followed by cooling at room temperature. Absorbance was estimated at 695 nm using a microplate reader. The assay was executed in triplicate, ascorbic acid was used a positive control and calibration curve was drawn (y = 0.0096x + 0.1538, R^2^ = 0.9972). Negative control used in the experiment was DMSO and results were expressed as μg ascorbic acid equivalent (AAE) per mg extract.

#### Determination of total reducing power (TRP)

Extracts of selected *Datura* species were assessed for their reducing power by slight modification of previously reported protocol [22]. Briefly, 200 μl of extracts (4 mg/ml DMSO) were mixed with 400 μl each of phosphate buffer (0.2 M, pH 6.6) and 1% w/v potassium ferricyanide [K3Fe (CN)6] and the mixture was incubated at 30 °C for 50 min. Trichloroacetic acid (400 μl of 10% w/v solution) was added to the mixture followed by centrifugation at 3000 rpm for 10 min at room temperature. Supernatant was collected and 150 μl of it was transferred to wells of a 96 well microplate. Finally, FeCl_3_ (50 μl of 0.1% w/v solution) was added to the corresponding wells and absorbance was measured at 700 nm. DMSO was used as negative control while ascorbic acid served as positive control at 6.25–100 μg/ml final concentration and calibration curve was drawn (y = 0.0254x – 0.0543, R^2^ = 0.9908). Estimated TRP was expressed as μg ascorbic acid equivalent (AAE) per mg extract.

#### DPPH free radical scavenging assay

Free radical scavenging potential of *Datura* extracts was evaluated by determining their ability to quench the stable 2, 2-diphenyl 1-picrylhydrazyl (DPPH) free radical [19]. The assay was performed in a 96 well plate and any discoloration of the purple colored DPPH solution indicated radical scavenging activity. An aliquot of extracts (20 μl from 4 mg/ml DMSO stock solution) was poured in respective wells of the 96 well plate followed by addition of 180 μl of DPPH solution to make the final volume and concentration equal to 200 μl and 400 μg/ml respectively. Positive control used in the assay was ascorbic acid (1 mg/ml) while DMSO was employed as a negative control. Following 30 min incubation at 37 °C, change in color was noted and absorbance was measured at 517 nm. Decline in absorbance of the reaction mixture manifest the free radical scavenging potential of extracts. Percent scavenging activity was determined by using the following formula:

$$ \% RSA=1-\frac{OD\  of\ sample}{OD\  of\ negative\ control} \times 100 $$

Where OD stands for optical density.

Samples which showed greater than 50% inhibition at 400 μg/ml were tested at lower concentrations and their IC_50_ values were estimated.

### In vitro cytotoxic assays

#### Brine shrimp lethality assay

Lethality of test samples against larvae of brine shrimps (*Artemia salina*) was evaluated in 96 well plate format following a previously reported protocol [23]. Simulated sea water (sea salt; 38 g/l supplemented with 6 mg/l dried yeast) was prepared and *Artemia salina* eggs (Ocean 90, USA) were kept therein for a period of 24–48 h with proper oxygen supply allowing them to hatch. Specifically designed perforated plastic tray was used having two compartments. Sources of illumination and warmth (30–32 °C) were also ensured. Phototropic nauplii were then shifted to respective wells of a 96 well plate. The cytotoxic activity of test samples was screened initially at 200, 100 and 50 μg/ml final concentration by transferring predetermined volume of extracts (20 mg/ml stock solution) to wells containing shrimps larvae and sea water. Doxorubicin (4 mg/ml) was used as a positive control while DMSO was the negative control. Following a 24 h incubation period, lethality of test samples were estimated by observing the number of surviving shrimps. Samples showing greater than 50% mortality were tested at lower concentrations and median lethal concentration (LC_50_) was calculated using table curve 2D v5.01 software. Surviving shrimps after completion of assay were treated with 6% sodium hypochlorite solution for 1 hour and disposed of in the septic tank. Surplus untested shrimps were used as fish food in dedicated fish pond at the University campus.

#### Protein kinase inhibition assay

*Stremtomyces* 85E was used as a test strain in the execution of this assay following an already reported protocol [24]. Spores of test strain were inoculated in sterile trypton soya broth (TSB). Incubation at 37 °C for 24 h was followed by turbidity adjustment as per McFarland 0.5 turbidity standard using sterile TSB. Petri plates with minimal ISP4 medium were seeded with inoculum of *Stremtomyces* (100 μl)*.* Sterile filter paper discs impregnated with extracts of selected *Datura* species (5 μl from 20 mg/ml DMSO stock solution) were positioned on the seeded agar plates. Positive and negative controls used were surfactin and DMSO respectively. Plates were incubated at 28 °C for a duration sufficient enough to allow hyphae formation (up to 72 h). Appearance of bald zone around the discs showed that extracts have inhibited spores and mycelia formation. Results were interpreted by measuring diameter of inhibitory zones to the nearest mm.

#### Cytotoxicity against cell lines

In vitro cytotoxicity of extracts against PC-3 (ATCC® CRL-1435), MDA-MB 231 (ATCC# HTB-26™) and MCF-7 (ATCC# HTB-22™) cancer cell lines was assessed by following an already reported protocol [23]. Selected cancer cell lines were grown in RPMI-1640 growth medium buffered with 2.2 g/l NaHCO_3_ and supplemented with 10% v/v heat inactivated foetal bovine serum (HIFBS) having pH 7.4. Cells were grown and kept in a humidified CO_2_ (5%) incubator at 37 °C. Extracts were tested at a final concentration of 20 μg/ml by adding 10 μl from the stock solution (dissolved in 1% DMSO in PBS) to wells of 96 well plate. Afterwards, 190 μl of cell suspension (seeding density of 1 × 10^4^ cells/ml) was transferred to respective wells. The 96 well plate was then kept in humidified CO_2_ (5%) incubator at 37 °C for a period of 72 h. It was followed by addition of 20 μl of pre-filter sterilized MTT solution (4 mg/ml in distilled H_2_O) to the wells and re-incubation at 37 °C for 4 h in identical conditions. Afterwards, a multichannel pipette was used for careful removal of supernatant from the wells without unsettling the colored formazan sediments. These sediments were then dissolved by addition of 100 μl of DMSO to each well and complete dissolution was ensured by keeping the plate aside for 1 h. Absorbance was measured at 540 nm using microplate reader. Doxorubicin was used a positive control while 1% DMSO in PBS was the negative control used in current study. Extracts that showed greater than 50% cell death at 20 μg/ml were analyzed at lower concentrations (10, 5, 2.5 and 1.25 μg/ml) and their IC_50_ values were calculated using table curve 2D v5.01 software. The experiment was run in triplicate.

Out of 32 crude extracts in four different solvents, ethyl acetate leaf extracts of *D. stramonium* (DSL-EA) and *D. inoxia* (DIL-EA) were selected for estimation of in vivo antileukmic activity. Extracts were standardized on the basis of their significant phenolics and flavonoids content as well as noteworthy antioxidant, protein kinase inhibitory and cytotoxic potential.

### Animal model

Fifty four (54) male Sprague Dawley rats aged 6–8 weeks weighing in range of ~ 150–250 g were used in current study. The animals were kept in aluminum cages with wood shavings as bedding at 25 ± 1 °C and air humidity of 45 ± 5% with a 12 h light/dark cycle. The bedding was changed regularly to avoid any infection and health hazards to study animals. Rats were provided standard laboratory feed and water ad libitum preceding their use in experiments.

### Experimental protocol

After a 1 week acclimatization period, rats were divided randomly into nine groups, each group consisting of six rats. Predetermined doses (high dose; HD and low dose; LD) of DSL-EA, DIL-EA extracts as well as standard drug dissolved in 10% DMSO, were administered orally using sterilized disposable oral gavage to specified groups. Total volume to be given orally was kept constant at 1 ml. Three controls were included in the study i.e. vehicle, disease and positive control groups. Treatments were given in the morning on alternate days.

Group I: Vehicle control (10% DMSO in water).

Group II: Disease control (0.2 ml Benzene, 1:10 in water for injection).

Group III: Positive control (Cyclophosphamide 10 mg/kg).

Group IV: DSL-LD (100 mg/kg, Preventive).

Group V: DSL-HD (200 mg/kg, Preventive).

Group VI: DIL-LD (100 mg/kg, Preventive).

Group VII: DIL-HD (200 mg/kg, Preventive).

Group VIII: DSL-HD (200 mg/kg, Treatment).

Group IX: DIL-HD (200 mg/kg, Treatment).

**Fig. 1:**
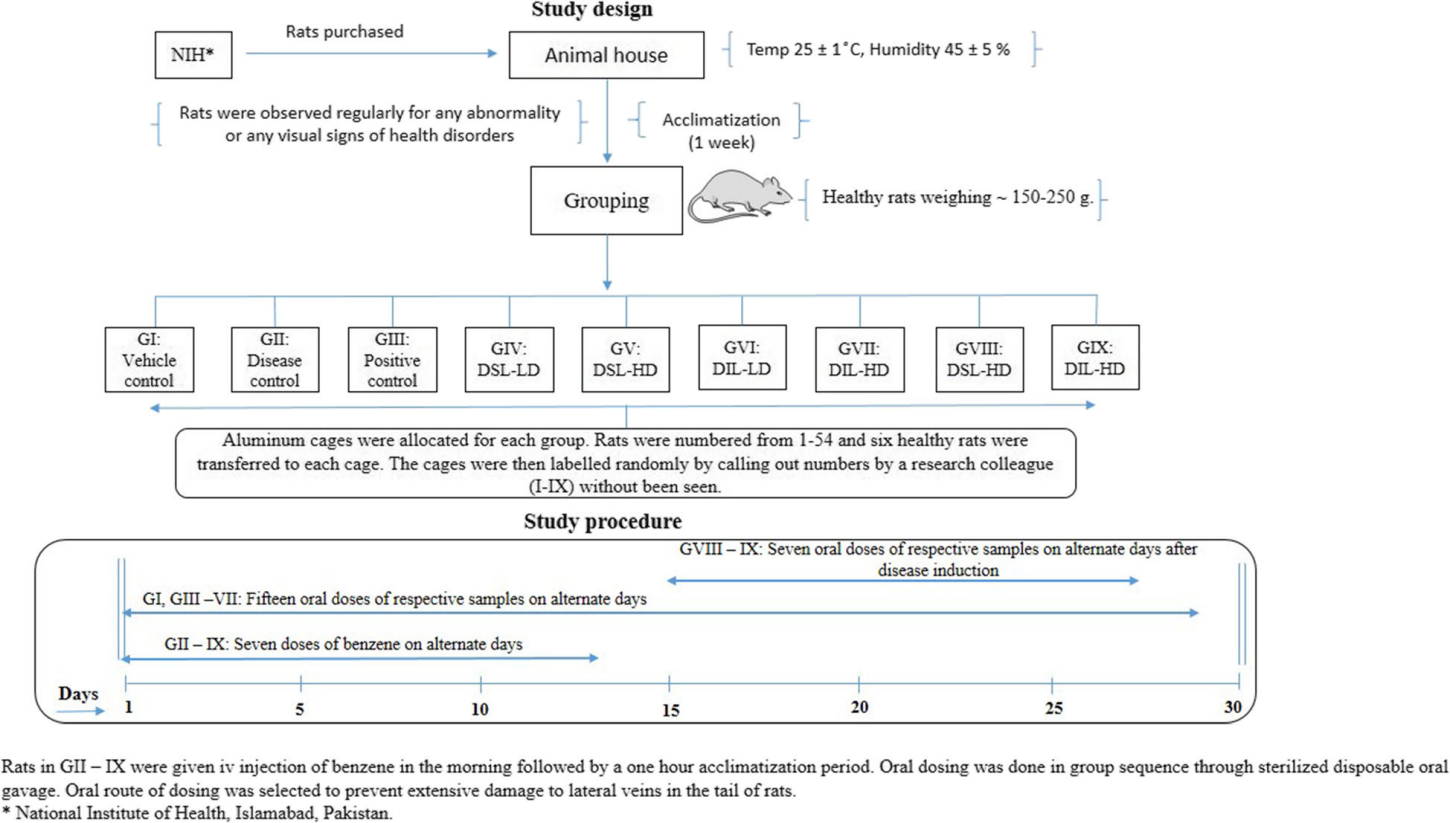
A schematic diagram of study design and experimental timeline of in vivo antileukemic activity performed using male Sprague Dawley rats. Clip art images used in the figure were made using ChemDraw Professional v19.0

The duration of the in vivo study was 30 days and groups in preventive mode (Group IV-VII) were orally administered with doses of *Datura* extracts starting from day one of experiment (15 doses). On the contrary, treatment groups (Group VIII and IX) received high doses of extracts after leukemia was induced in rats (7 doses). The details of study design and procedure are given in the schematic diagram Fig. 1.

### Animal ethical statement

The guidelines approved by the ethical committee of Quaid-i-Azam University, Islamabad, Pakistan (Letter No. QAU-PHM-023/2016 for experimentation and Letter No. QAU-PHM-017/2016 for the animal care) were strictly followed throughout the experiment. Blood sampling from healthy volunteers was also approved by review board of Quaid-i-Azam University (Letter No. IRB-QAU-116; Dated 4/11/2016). It was assured that the animals used in the study were handled with minimum possible discomfort, pain and distress by provision of appropriate analgesia, sedation and anesthesia. Animal/human use was also meant to offer insights and new information for the wellbeing of humans/animals.

### Toxicity assessment

#### In vitro toxicity testing by using isolated lymphocytes

Lymphocytes were isolated from human blood following a procedure reported earlier with modifications made as per system suitability [25]. A written informed consent was obtained from the participant. Blood (3 ml) was taken through venipuncture from a healthy donor and diluted with an equal portion of PBS. The diluted blood sample was layered over 2 ml Histopaque-1077 and centrifuged at 800×g for 20 min. The buffy coat was then aspirated into PBS (5 ml) and lymphocytes pellet was obtained by centrifugation at 350 rpm for 4 min. The pellet acquired was suspended in RPMI-1640 (1 ml) and cell density was adjusted to 1 × 10^5^ cells/ml. Cytotoxicity was determined by incubating 20 μl of extracts (20 μg/ml) along with 180 μl of lymphocyte suspension at 37 °C for 24 h in a 96 well plate. Humidified carbon dioxide (5%) incubator was used for incubation. Lymphocyte growth was stimulated by addition of phytohaemagglutinin (PHA) in the medium. Following the predetermined time period, MTT assay was performed as discussed above and percent mortality was calculated using the following formula:

$$ \% cytotoxicity=\left[100-\left(\frac{As}{Ac}\times 100\right)\right] $$

Where, As and Ac denote absorbance of sample and negative control respectively.

#### In vivo acute toxicity in rats

Acute toxicity assay was performed in rats divided randomly into test and control groups (*n* = 6). Rats in the test groups were orally administered with increasing doses of test samples (150, 300, 500, 1000 and 2000 mg/kg). Single booster dose of the mentioned strengths were given to respective groups while rats in the control group were given normal saline (10 ml/kg of animals). Animals were observed daily for a period of two weeks and mortality rates as well as toxic symptoms were noted. Any aberrations in behavioral pattern i.e. balance, aggression and sleep; body secretions i.e. lacrimation, salivation, nasal discharge, urination and defecation, as well as visual signs of any injury to different organs were closely monitored. Guidelines provided by Organization for Economic Cooperation and Development (OECD) were followed and ensured during acute toxicity study.

### In vivo antileukemic assay in rats

#### Benzene induced leukemia in rats

Rats used for the assay were divided randomly into 9 groups as described earlier and benzene model was adopted for leukemia induction [11, 26, 27]. Benzene (0.2 ml) was injected in the lateral tail vein after restraining the rat in a physical restrainer and keeping its tail in warm water (45 °C). Benzene was administered on alternate days for 2 weeks. Rats were observed for any visible signs of disease induction i.e. excessive bruising, unusual bleeding, dark colored urine, labored and/or rapid breathing, increased heart rate, fatigue, weakness, petechiae and skin rashes.

### Collection of blood samples and separation of serum

Rats were anesthetized at the end of the assay by chloroform inhalation, euthanized using cervical dislocation and blood was drawn from abdominal aorta of each rat for assessment of primary outcomes like hematological effects as well as secondary outcomes including relevant biochemical and serological findings. Centrifugation of blood samples at 6000 rpm (for 15 min at 4 °C) was performed for separation of serum. Serum was kept at − 20 °C until analyzed.

### Haematological studies

These involved the estimation of red blood cell (RBC), white blood cell (WBC) and platelet count with the help of neubauer haemocytometer (Feinoptik, Germany). Erythrocyte sedimentation rate (ESR) was estimated following Westergren method [28], while hemoglobin (Hb) content was determined by Sahli’s haemoglobin meter.

### Determination of biochemical parameters

Sera of experimental rats was analyzed for numerous biochemical parameters i.e. alanine transaminase (ALT), aspartate aminotransferase (AST), alkaline phosphatase (ALP), creatinine phosphokinase (CPK), total serum protein, urea and creatinine using standard AMP diagnostic kits (Stattogger Strasse 31b 8045 Graz, Austria) while Bradford method was followed for the determination of protein concentration [29].

### Determination of endogenous antioxidant enzymes

For the assessment of endogenous antioxidant enzymes i.e. catalase (CAT), peroxidase (POD), superoxide dismutase (SOD) and glutathione S-transferase (GST), serum separated from the blood of rats was used and an already reported protocol [30] was followed with slight modifications as per system suitability.

### Expression of TBARs and NO

Level of Thiobarbituric acid reactive substances (TBARs) in serum was estimated by TBARs assay using a slightly modified version of an already described procedure [30].

Nitric oxide (NO) levels were calculated using procedure described by Grisham et al.*,* [31]. Following a slightly modified protocol, serum (50 μl) was mixed with equal portions of 0.3 M NaOH and 5% ZnSO4. Centrifugation was performed at 6400×g for 15–20 min. Supernatant (30 μl) was collected, transferred to 96 well plate and mixed with 200 μl of Griess reagent. Absorbance was recorded at 540 nm. Sodium nitrite curve (y = 0.0015x + 0.002, R^2^ = 0.9821) was drawn under identical conditions to estimate serum nitrite level.

### Histological investigation

Paraffin embedded staining procedure was employed for evaluation of any changes in normal histology. Dissected tissues of liver and kidney were fixed with the help of buffered formaldehyde (10%, pH 7.4) at room temperature for a period of 12 h. Fixed tissues were washed repeatedly with ethanol (50, 70, 90 and 100%) to remove any traces of infiltrated wax and water. Slides were prepared by sectioning small pieces (3–5 μm thickness) of the embedded tissue samples which were then stained with Eosin [31] and Haematoxylin. Subsequently, slides were examined under Nikon Microscope (Eclipse 80i, Japan).

### Percent alleviative effects of *Datura* extracts in benzene induced leukemia

Antileukemic potential of both species was evaluated by determining the alleviative effects in comparison to the disease control group which did not receive any treatment. Percent antileukemic effects produced by *Datura* species were statistically compared with the results procured in case of positive control group. Percent alleviating/antileukemic potential was evaluated by using the following formula:

$$ \% Alleviating\ effects=\frac{average\ sample\ values\kern0.5em - average\ disease\ control\ values\ }{average\ disease\ control\ values}\times 100 $$

### Statistical analysis

The data presented in this study was procured from experiments performed in triplicate and values are presented as mean ± SD. Variability amongst groups was determined by performing one way analysis of variance using Statistix 8.1. Tukey’s multiple comparison and Kruskal-Wallis tests were performed to calculate significant differences among test groups. Statistical significance was set at *p* < 0.05.

## Results

Ethyl acetate leaf extracts of *D. stramonium* (DSL-EA) and *D. inoxia* (DIL-EA) were selected based on proficient results in preliminary in vitro screening assays.

### Percent extract recovery

Percent extract recovery in case of DSL-EA extract was 2.33% while it was 2.59% in case of DIL-EA extract.

### Phytochemical investigation

#### Total phenolic and flavonoid content

Phenolic and flavonoid content of EA leaf extracts of both plants was estimated. TPC estimated in DSL-EA was 28.67 ± 0.97, while in case of DIL-EA it was 27.69 ± 1.12 μg GAE/mg extract. Quercetin equivalent flavonoid content estimated in DSL-EA was 16.16 ± 0.06 and in DIL-EA the content was 20.17 ± 0.17 μg QE/mg extract as presented inTable 1.

**Table 1:** Total phenolic and flavonoid content of EA leaf extracts of *D.stramonium* and *D.inoxia*

Samples	TPC (μg GAE/mg extract)	TFC (μg QE/mg extract)
DSL-EA	28.67 ± 0.97	16.16 ± 0.06
DIL-EA	27.69 ± 1.12	20.17 ± 0.17

#### RP HPLC analysis

**Fig. 2:**
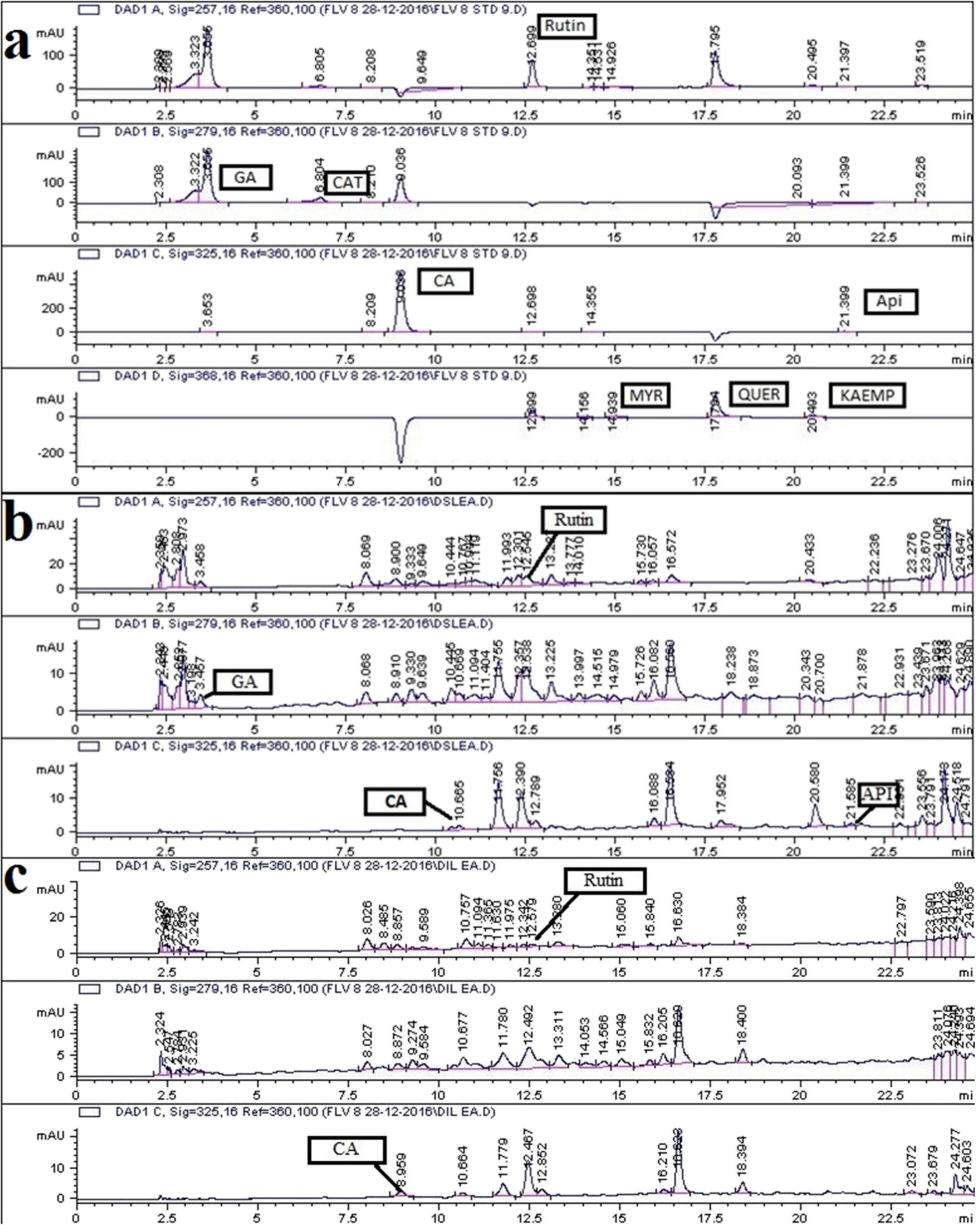
**a** RP-HPLC Chromatogram of standard polyphenols. **b** Chromatogram of compounds detected in ethyl acetate leaf extract of *D. stramonium* and **c** Chromatogram of compounds detected in ethyl acetate leaf extract *D. inoxia*. API- Apigenin, CA- Caffeic acid, CAT- Catechin, GA- Gallic acid, KAEMP- Kaempferol, MYR- Myricetin and QUER- Quercetin

Quantification of selected plant phenolics was accomplished with the aid of reverse phase HPLC-DAD based profiling. Retention time and UV spectra of extracts were compared with reference standards. Figure 2 and Additional file 1-3. Rutin, gallic acid, catechin, apigenin and caffeic acid were quantified in the tested extracts. In DSL-EA extract rutin (0.89 ± 0.025 μg/mg extract), gallic acid (0.35 ± 0.072 μg/mg extract), catechin (0.24 ± 0.023 μg/mg extract) and apigenin (0.29 ± 0.091 μg/mg extract) were detected. Rutin (0.036 ± 0.004) and caffeic acid (0.27 ± 0.031 μg/mg extract) was quantified in DIL-EA. (Table 2).

**Table 2:** Chemical profiling of EA leaf extracts of *D.stramonium* and *D.inoxia* using HPLC-DAD

Polyphenols	Signal wavelength	Quantity (μg/mg extract)	
		DSL-EA	DIL-EA
Rutin	257	0.89 ± 0.03^***^	0.036 ± 0.004^*^
Gallic acid	279	0.35 ± 0.07^**^	–
Catechin	279	0.24 ± 0.02^**^	–
Caffeic acid	325	–	0.27 ± 0.03**
Apigenin	325	0.29 ± 0.09^**^	–
Myricetin	368	–	–
Quercetin	368	–	–
Kaempferol	368	–	–

*** = significant, ** = fair, and * = slight concentration

### In vitro antioxidant potential

#### Total antioxidant capacity and total reducing power

The total antioxidant capacity of extracts was appraised using phosphomolybdenum based method and results were expressed as ascorbic acid equivalent (AAE). TAC exhibited by DSL-EA was 160.92 ± 3.00 μg AAE/mg extract, while in case of DIL-EA it was 140.44 ± 5.6 μg AAE/mg extract (Table 3).

**Table 3:** In vitro antioxidant potential of EA leaf extracts of *D.stramonium* and *D.inoxia*

Samples	TAC (μg AAE/mg extract	TRP (μg AAE/mg extract	DPPH (% scavenging at 400 μg/ml)
DSL-EA	160.92 ± 3.00	50.43 ± 1.72	< 50%
DIL-EA	140.44 ± 5.6	44.02 ± 1.30	< 50%

TRP was manifested by evaluating the ferric ion (Fe^3+^) conversion to ferrous ion (Fe^2+^) in the presence of known concentration of extracts. Results indicated that reducing power depicted by DSL-EA was 50.43 ± 1.72 μg AAE/mg extract, while DIL-EA followed with 44.02 ± 1.30 μg AAE/mg extract (Table 3).

#### DPPH scavenging assay

Extracts were tested for their percent free radical scavenging activity (% FRSA), both DSL-EA and DIL-EA did not show any appreciable scavenging potential as shown in Table 3.

### In vitro cytotoxicity assessment

#### Brine shrimps cytotoxicity assay

Cytotoxicity profile was evaluated with the aid of brine shrimp lethality assay. Selected extracts of both *Datura* species showed remarkable activity. DSL-EA and DIL-EA showed significant results with LC_50_ values of 12.04 ± 0.85 and 10.37 ± 1.56 μg/ml respectively. Doxorubicin was used as a standard and its LC_50_ value was recorded to be 5.43 μg/ml. Results are shown in Table 4.

**Table 4:** Cytotoxicity and protein kinase inhibition of DSL-EA and DIL-EA extracts

Samples	Brine shrimp cytotoxicity		Protein kinase inhibition			Cytotoxicity							
						PC-3		MDA-MB 231		MCF-7		Normal lymphocytes	
	% Mortality 200 μg/ml	LC_50_ μg/ml	Diameter at 100 μg/disc		MIC μg/disc	% inhibition 20 μg/ml	IC_50_ μg/ml	% Inhibition 20 μg/ml	IC_50_ μg/ml	% Inhibition 20 μg/ml	IC_50_ μg/ml	% Inhibition 20 μg/ml	IC_50_ μg/ml
			Clear zone	Bald zone									
DSL-EA	96.96 ± 5.25^a^	12.04 ± 0.21	7.5 ± 0.58	12.50 ± 0.58^c^	100	14.4 ± 0.4^b^	–	38.03 ± 0.38^b^	–	40.2 ± 0.3^b^	–	4 ± 0.35^c^	–
DIL-EA	100 ± 0.00^a^	10.37 ± 0.56	9.00 ± 1	19.00 ± 0.58^b^	25	99.93 ± 0.11^a^	2.86 ± 0.1	98.64 ± 0.33^a^	1.56 ± 0.16	99.36 ± 0.72^a^	2.45 ± 0.04	23 ± 1.45^b^	–
Vincristine	–	–	–	–	–	–	–	–	–	–	–	79 ± 2.55^a^	6.89 ± 0.19
Doxorubicin	100 ± 0.00^a^	5.74 ± 0.18	–	–	–	99.43 ± 0.15^a^	2.95 ± 0.11	98.11 ± 0.48^a^	2.45 ± 0.24	98.62 ± 0.42^a^	3.2 ± 0.11	–	–
Surfactin	–	–	–	26.50 ± 1.0^a^	–	–	–	–	–	–	–	–	–
DMSO	–	–	–	–	–	–	–	–	–	–	–	–	–

Values are presented as mean ± SD of triplicate analysis. Results of standards and/or controls are given in respective columns. Means with different superscripts (a-c) represent significantly different values at *p < 0.05*

#### Protein kinase inhibition assay

The capability of extracts to inhibit protein kinase and subsequently the aerial hyphae formation by extracts was gauged on the basis of appearance of bald zones on cultured plates. DIL-EA showed significant inhibitory potential by exhibiting a 19 mm bald phenotype zone at a concentration of 100 μg/disc (MIC: 25 μg/disc). Moreover, DSL-EA exhibited a bald zone of 12.50 mm at the same concentration (MIC: 100 μg/disc). Surfactin been used as a positive control exhibited a 26.5 mm zone while DMSO (negative control) did not show any inhibitory potential. The results are demonstrated in Table 4.

#### Cytotoxicity against cell lines

Cytotoxicity against PC-3, MDA-MB 231 and MCF-7 was determined initially as percent inhibition at 20 μg/ml final concentration followed by determination of IC_50_ values of potent extracts. Details are given in Table 4. The most promising activity was shown by DIL-EA against each cancer cell line with IC_50_ values of 2.86 ± 0.1 μg/ml against PC-3, 1.56 ± 0.16 μg/ml against MDA-MB 231 and 2.45 ± 0.04 μg/ml in case of MCF-7 cell line. DSL-EA showed mediocre cytotoxic potential with 14.34% inhibition against PC-3, 37.67% in case of MDA-MB 231 and 40.56% against MCF-7 cell line. Doxorubicin was the positive control used and it exhibited IC_50_ values of 2.95 ± 0.1, 3.2 ± 0.11 and 2.45 ± 0.24 μg/ml against PC-3, MDA-MB 231 and MCF-7 cell lines respectively.

### Toxicity studies

#### Lymphocyte toxicity assay

Cytotoxic nature of selected extracts was also evaluated against normal lymphocytes isolated from human blood. Percent inhibition was calculated at 20 μg/ml final concentration (Table 4). No significant activity was depicted by either DSL-EA or DIL-EA against normal lymphocytes. The cytotoxicity of DS-L-EA and DIL-EA was merely 4 and 23% respectively. Observed cytotoxicity was significantly (*p* < 0.05) lower in comparison to that observed against cancer cells. This selective action of used extracts is extremely beneficial in targeting cancerous cells while sparing the normal ones. Vincristine was used as a positive control and it exhibited an IC_50_ value of 6.98 ± 0.19 μg/ml.

#### In vivo acute toxicity assay

Acute toxicity was evaluated in rats at doses of 150–2000 mg/kg. Close surveillance of rats during an observation period of 2 weeks showed that DSL-EA and DIL-EA had no lethal effects. There were no notable behavioral changes nor any deaths witnessed during the said period. Rats showed no visible signs of abnormalities in senses and any deviations from normal physiology. These findings showed that the chosen extracts were safe up to the highest dose of 2000 mg/kg and additional pharmacological studies can be safely undertaken within the specified range.

### In vivo antileukemic assay

Following intravenous administration of predetermined doses of benzene to preventive, treatment and disease control groups, obvious signs of leukemia induction were observed including, unusual bleeding, excessive bruising, skin rashes, weakness and weight loss. Efficacy of extracts in treatment or prevention of disease progression was measured through a series of haematological, biochemical and histological studies.

### Benzene induced haematological variations

Results of benzene induced haematological variations are displayed in Table 5. As evident from the data, the carcinogen used has caused a whole lot haematological aberrations, most eminent of which are decline in RBCs (4.33 ± 0.065 × 10^6^/μl), platelets (344 ± 3.19 × 10^3^/μl) and haemoglobin levels (5.9 ± 0.26 g/dl) while increase in ESR (9.6 ± 0.12 mm/h) and WBCs count (7.78 ± 0.012 × 10^3^/μl) was also observed in disease control rats. Findings of hematological investigation revealed that the positive control, vehicle control, preventive and treatment groups are significantly different (*p* < 0.05) from the disease control group. The average RBC and platelets count in preventive and treatment groups of both plant species were raised up to 5.78 ± 0.23 × 10^6^/μl and 529 ± 20.17 × 10^3^/μl respectively, while average haemoglobin levels were elevated to 7.43 ± 0.25 g/dl. High and low doses of both species of *Datura* normalized the WBCs count and ESR with average values of 3.95 ± 0.26 × 10^3^/μl and 6.08 ± 0.24 mm/h respectively.

**Table 5:** Haematological investigations of experimental rats of all study groups

Groups	RBCs (× 10^6^)/μl	WBCs (× 10^3^)/μl	Platelets (× 10^3^) /μl	Hb (g/dl)	ESR (mm/h)
Vehicle control	6.23 ± 0.035^a^	3.55 ± 0.018^f^	581 ± 2.72^a^	9.78 ± 0.62^a^	4.4 ± 0.067^g^
Positive control	6.03 ± 0.095^a^	3.85 ± 0.067^d^	495 ± 2.72^e^	8.38 ± 0.62^b^	5.4 ± 0.067^f^
Disease control	4.33 ± 0.065^c^	7.78 ± 0.012^a^	344 ± 3.19^f^	5.9 ± 0.26^e^	9.6 ± 0.12^a^
DSL LD (P)	5.73 ± 0.071^ab^	3.61 ± 0.025^de^	538 ± 1.98^c^	7.7 ± 0.35^cd^	5.7 ± 0.071^e^
DSL HD (P)	5.95 ± 0.083^ab^	3.72 ± 0.038^de^	556 ± 2.59^b^	7.7 ± 0.47^bc^	5.9 ± 0.066^d^
DIL LD (P)	5.86 ± 0.037^ab^	3.86 ± 0.027^d^	523 ± 3.07^d^	7.34 ± 0.72^cd^	6.2 ± 0.034^c^
DIL HD (P)	6.02 ± 0.065^a^	4.01 ± 0.011^c^	541 ± 2.63^c^	7.12 ± 0.58^cd^	6.12 ± 0.081^c^
DSL HD (T)	5.36 ± 0.069^b^	4.23 ± 0.044^b^	517 ± 1.98^d^	7.2 ± 0.28^d^	6.21 ± 0.036^c^
DIL HD (T)	5.73 ± 0.081^b^	4.25 ± 0.035^b^	499 ± 2.94^e^	7.52 ± 0.79^cd^	6.34 ± 0.097^b^

Values are presented as mean ± SD (*n* = 6, where n = number of rats analysed in each group). Means with different superscripts (a-g) in the columns are significantly (p < 0.05) different from one another

### Determination of enzymatic and biochemical parameters

Various enzymatic and biochemical tests performed on serum acquired from rats of study groups have clearly shown the deleterious effects of benzene on vital organs including liver and kidney in the disease control group. Administration of low and high doses of DSL-EA and DIL-EA have curbed the harm instigated by benzene to a great extent as evident from the enzymatic and biochemical findings of test groups. Liver enzymes and CPK levels of disease control group were significantly higher (*p* < 0.05) than all other groups. ALT, AST, ALP and CPK levels estimated in disease control rats were 93.02 ± 2.64, 67.80 ± 2.05, 356 ± 5.69 and 234 ± 4.97 U/L respectively. Moreover, total proteins estimated in the serum were expressively lower in case of disease control rats (Albumin 1.20 ± 0.07, globulin 0.94 ± 0.07 and total protein 2.14 ± 0.11 g/dl) confirming the harm caused to liver by benzene. Low and high doses of tested extracts have reverted the liver damage in identical manner, average values of ALT, AST, ALP, CPK and total proteins of 6 groups (both preventive and treatment) are; 41.56 ± 2.59, 27.99 ± 1.98. 141 ± 8.46, 148.1 ± 7.98 U/L and 6.54 ± 0.38 g/dl respectively. The details are given in Tables 6 and 7.

**Table 6:** Enzymatic investigation of control and leukemic rats

Groups	ALT (U/L)	AST (U/L)	ALP (U/L)	CPK (U/L)
Vehicle control	41.3 ± 1.65^e^	23 ± 0.26^g^	127 ± 3.28^b^	133 ± 4.04^g^
Positive control	47.3 ± 1.87^b^	28 ± 0.63^d^	143 ± 3.57^b^	139 ± 3.28^ef^
Disease control	93.20 ± 2.64^a^	67.80 ± 2.05^a^	356 ± 5.69^a^	234 ± 4.97^a^
DSL LD (P)	45.31 ± 2.28^c^	24.65 ± 1.01^f^	148 ± 3.26^b^	154 ± 2.32^c^
DSL HD (P)	42.54 ± 1.79^d^	26.81 ± 0.86^e^	139 ± 4.02^b^	146 ± 2.86^d^
DIL LD (P)	42.81 ± 1.66^d^	29.34 ± 1.28^c^	152 ± 3.89^b^	159 ± 3.47^b^
DIL HD (P)	40.76 ± 2.26^e^	28.11 ± 0.94^d^	143 ± 2.93^b^	151 ± 3.63^c^
DSL HD (T)	39.64 ± 1.95^f^	29.06 ± 0.68^c^	135 ± 3.61^b^	141 ± 2.79^e^
DIL HD (T)	38.32 ± 1.58^g^	30.01 ± 1.14^b^	129 ± 4.01^b^	138 ± 3.34^f^

Results are represented as mean ± SD (n = 6, where n = number of rats analysed in each group). Mean values with different superscripts (a-g) in the columns are significantly (p < 0.05) different from one another

**Table 7:** Biochemical investigation of control and leukemic rats

Groups	Urea (mg/dl)	Creatinine (mg/dl)	Bilirubin (mg/ml)	Albumin (g/dl)	Globulin (g/dl)	Total protein (g/dl)
Vehicle control	29 ± 1.76^g^	1.01 ± 0.11^b^	2.7 ± 0.17^e^	3.9 ± 0.18^c^	2.8 ± 0.13^b^	6.70 ± 0.32^c^
Positive control	26 ± 1.01^h^	1.08 ± 0.14^b^	2.67 ± 0.21^e^	4.07 ± 0.24^c^	2.79 ± 0.16^b^	6.86 ± 0.38^b^
Disease control	72.2 ± 2.76^a^	2.89 ± 0.54^a^	7.56 ± 0.97^a^	1.20 ± 0.07^e^	0.94 ± 0.07^g^	2.14 ± 0.11^f^
DSL LD (P)	36.21 ± 0.98^b^	1.19 ± 0.09^b^	3.21 ± 0.09^b^	4.56 ± 0.15^a^	2.05 ± 0.09^f^	6.61 ± 0.53^c^
DSL HD (P)	32.34 ± 1.76^e^	1.06 ± 0.12^b^	2.89 ± 0.64^d^	4.01 ± 0.21^c^	2.98 ± 0.18^a^	6.99 ± 0.59^a^
DIL LD (P)	35.14 ± 0.85^c^	1.12 ± 0.11^b^	3.04 ± 0.33^c^	4.32 ± 0.19^b^	2.56 ± 0.15^c^	6.88 ± 0.63^ab^
DIL HD (P)	34.12 ± 1.23^d^	1.08 ± 0.08^b^	2.98 ± 0.26^c^	4.27 ± 0.26^b^	2.32 ± 0.13^d^	6.59 ± 0.49^c^
DSL HD (T)	30.09 ± 2.05^f^	1.01 ± 0.10^b^	2.48 ± 0.08^f^	3.64 ± 0.17^d^	2.39 ± 0.08^d^	6.03 ± 0.37^e^
DIL HD (T)	28.20 ± 1.87^g^	1.02 ± 0.07^b^	2.42 ± 0.39^f^	3.96 ± 0.23^c^	2.19 ± 0.11^e^	6.15 ± 0.42^d^

Values are represented as mean ± SD (*n* = 6, where n = number of rats analysed in each group). Means with different superscripts (a-h) in the columns are significantly (p < 0.05) different from one another

Creatinine, urea and bilirubin levels were markedly high in disease control rats confirming acute leukemic condition and kidney damage. High and low doses of selected plants have notably reversed the abuse inflicted by benzene as evident in Table 7. Statistically significant difference (*p* < 0.05) was observed when positive control and other groups were compared with disease control group.

### Endogenous antioxidant enzymes

The activity level of endogenous antioxidant enzymes in serum is presented in Table 8. A significant (*p* < 0.05) decline in CAT, POD, SOD and GST was estimated in disease control group when compared with vehicle and positive control groups. Activity levels of CAT, POD, SOD and GST in serum drawn from disease control rats were 0.5 ± 0.04, 1.00 ± 0.04 U/min, 0.96 ± 0.05 U/mg protein and 81.01 ± 0.44 nM/min/ml respectively. A dose dependent increase in CAT, SOD, POD and GST level was observed in test groups. Significant (*p* < 0.05) increase was observed in case of high doses of selected plants in preventive and treatment groups. High doses of DIL raised the activity levels of CAT, POD, SOD and GST to a reasonably greater extent as compared to similar doses of DSL in treatment mode i.e. 2.5 ± 0.15, 3.9 ± 0.10 U/min, 2.27 ± 0.09 U/mg protein and 162.03 ± 1.54 nM/min/ml respectively. Low doses given to preventive groups showed slight increase in antioxidant enzymes and GST level.

**Table 8:** Effect of *Datura* extracts on activity levels of endogenous antioxidant enzymes

Groups	CAT (U/min)	POD (U/min)	SOD (U/mg protein)	GST (nM/min/ml)
Vehicle control	1.7 ± 0.02^c^	2.1 ± 0.03^e^	4.00 ± 0.05^a^	216.04 ± 1.46^c^
Positive control	2.2 ± 0.04^b^	3.1 ± 0.06^c^	3.51 ± 0.03^b^	236.04 ± 1.46^b^
Disease control	0.5 ± 0.04^e^	1 ± 0.04^g^	0.96 ± 0.05^g^	81.01 ± 0.44^g^
DSL LD (P)	0.9 ± 0.07^d^	1.7 ± 0.02^f^	2.42 ± 0.02^f^	108.03 ± 0.63^f^
DSL HD (P)	1 ± 0.006^d^	2.2 ± 0.01^e^	2.83 ± 0.04^d^	162.02 ± 0.70^d^
DIL LD (P)	1.8 ± 0.06^c^	1.6 ± 0.04^f^	2.79 ± 0.02^d^	108.02 ± 1.03^f^
DIL HD (P)	2.0 ± 0.13^b^	4.2 ± 0.03^a^	3.18 ± 0.03^c^	297.05 ± 1.60^a^
DSL HD (T)	2.1 ± 0.08^b^	2.8 ± 0.09^d^	2.65 ± 0.10^e^	135.02 ± 0.63^e^
DIL HD (T)	2.5 ± 0.15^a^	3.9 ± 0.10^b^	2.72 ± 0.09^e^	162.03 ± 1.54^d^

Results are expressed as mean ± SD (*n* = 6, where *n* = number of rats analysed in each group). Values with different superscripts (a-g) in the columns indicate they are significantly (*p* < 0.05) different form each other

### Expression of TBARs and NO

After completion of the assay, TBARs and NO levels were recorded in serum obtained from test rats of each group. Benzene treated disease control group showed escalated level of TBARs i.e. 133.75 ± 2.61 nM/min/mg protein. Treatment groups of DSL-EA and DIL-EA have significantly (*p* < 0.05) lowered the level of TBARs i.e. 83.49 ± and 92.25 ± 1.17 nM/min/mg protein respectively. Estimated NO level was markedly high in disease control group with value 93.81 ± 2.88 μM/ml while positive control group showed significant decrease in NO level i.e. 61.74 ± 1.98 μM/ml. Low and high doses of DSL-EA and DIL-EA exhibited expressively reduced NO levels compared to disease control group with values not higher than 65 μM/ml in any of the study groups. Results are given in Table 9.

**Table 9:** TBARs and NO estimation of controls and leukemic rats

Groups	TBARs (nM/min/mg protein)	NO (μM/ml)
Vehicle control	88.90 ± 1.87^f^	60.74 ± 2.23^e^
Positive control	85.80 ± 1.09^g^	61.74 ± 1.98^d^
Disease control	133.75 ± 2.61^a^	93.81 ± 2.88^a^
DSL LD (P)	113.77 ± 2.21^b^	60.56 ± 1.62^e^
DSL HD (P)	100.82 ± 1.48^d^	62.52 ± 2.25^c^
DIL LD (P)	113.12 ± 2.32^b^	62.81 ± 2.73^c^
DIL HD (P)	105.82 ± 1.62^c^	64.89 ± 2.64^b^
DSL HD (T)	83.49 ± 1.19^h^	62.22 ± 1.76^c^
DIL HD (T)	92.25 ± 1.77^e^	63.41 ± 1.59^b^

Results are expressed as mean ± SD (*n* = 6, where *n* = number of rats analysed in each group). Mean values with different superscripts (a-h) in individual columns specify significance at *p < 0.05*

### Histological investigation

**Fig. 3:**
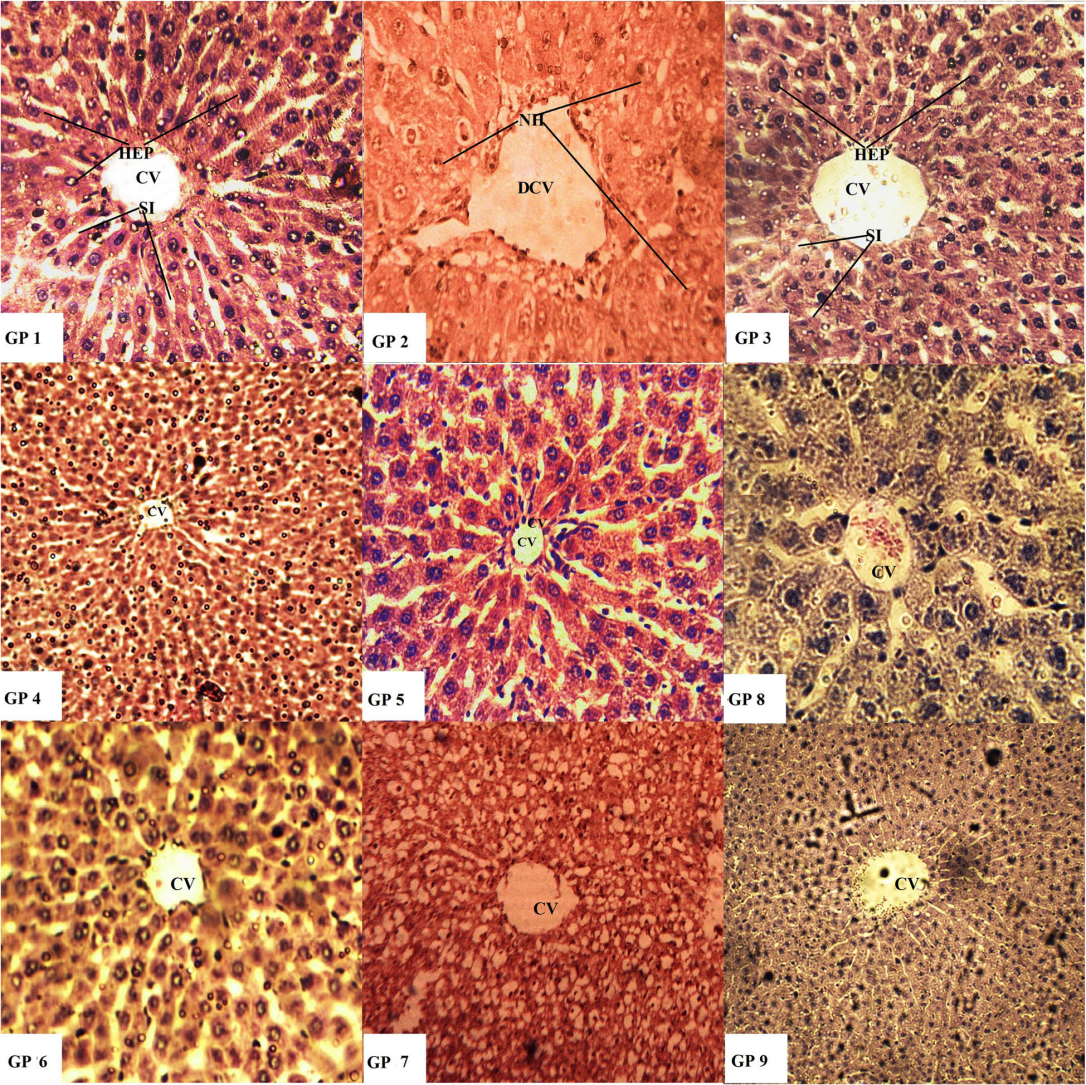
Hematoxylin-eosin stain. Histopathological observations (40X) for the protective potential of ethyl acetate extracts of *D.stramonium* and *D. inoxia* on liver in rat. Gp I: Vehicle control (10% DMSO in water), Gp II: Disease control (0.2 ml Benzene), Gp III:Positive control (Cyclophosphamide 10 mg/kg), Gp IV: DSL-LD (100 mg/kg, Preventive), Gp V: DSL-HD (200 mg/kg, Preventive), Gp VI: DIL-LD (100 mg/kg, Preventive), Gp VII: DIL-HD (200 mg/kg, Preventive), Gp VIII: DSL-HD (200 mg/kg, Treatment) and Gp IX: DIL-HD (200 mg/kg, Treatment). CV- Central vein, DCV- Damaged central vein, HEP- Hepatocytes, NH- Necrosed hepatocytes

**Fig. 4:**
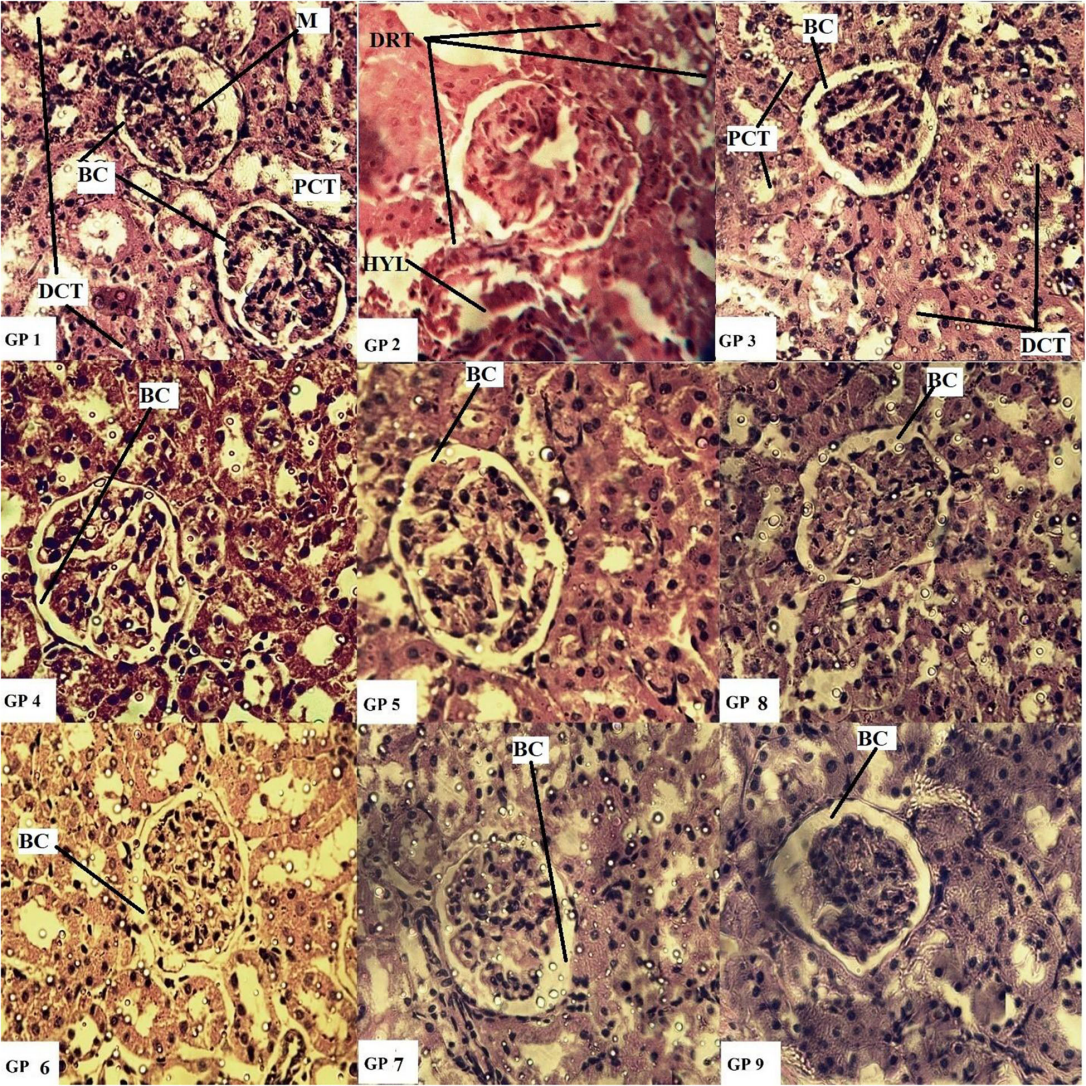
Hematoxylin-eosin stain. Histopathological observations (40X) for the protective potential of ethyl acetate extracts of *D.stramonium* and *D. inoxia* on kidney in rat. Gp I: Vehicle control (10% DMSO in water), Gp II: Disease control (0.2 ml Benzene), Gp III:Positive control (Cyclophosphamide 10 mg/kg), Gp IV: DSL-LD (100 mg/kg, Preventive), Gp V: DSL-HD (200 mg/kg, Preventive), Gp VI: DIL-LD (100 mg/kg, Preventive), Gp VII: DIL-HD (200 mg/kg, Preventive), Gp VIII: DSL-HD (200 mg/kg, Treatment) and Gp IX: DIL-HD (200 mg/kg, Treatment). BC- Bowman’s capsule, DCT: Distal convoluted tubules, DRT- Degenerated renal tubules, HYL- Hylinization (of glomerular tuft), M- Mesangium, PCT- Proximal convoluted tubules

Histological investigations have verified and endorsed the effects of EA extracts of selected *Datura* species on the studied biochemical parameters (particularly LFTs). Liver and kidney of the vehicle and positive control groups (group I and III) exhibited normal morphological features i.e. intact hepatocytes, sinusoids, typical central veins, Bowman’s capsule and glomerular tuft. Benzene treated disease control group caused significant damage to liver and kidney tissues i.e. necrosed hepatocytes, cellular hypertrophy, hylinization of glomerular tuft and marked degeneration of renal tubules as evident in Figs. 3 and 4 (10X images in Additional files 4-21). Low and high doses of administered extracts alleviated benzene induced damage to a greater extent as evident in the figure (Group IV-IX). These images further reinforce the findings of biochemical and enzymatic parameters.

### Percent alleviative effects of *Datura* extracts in benzene induced leukemia

The antileukemic effects of DSL-EA and DIL-EA are summarized in Table 10. Percent alleviating effects of both extracts against benzene induced leukemia are collectively illustrated in the table. Apart from the activity levels of antioxidant enzymes i.e. CAT, POD, SOD and GST, where DIL-EA exhibited an expressively greater curative effect, all other parameters were stabilized in a comparative and significant manner by both extracts.

**Table 10:** Percent alleviative effects of DSL-EA and DIL-EA extracts in benzene induced leukemia

Evaluation parameters	Study Groups							
	Disease control	Positive control	DSL-LD (P)	DSL-HD (P)	DIL LD (P)	DIL HD (P)	DSL HD (T)	DIL HD (T)
		Percent alleviative effect						
Haematalogical parameters								
RBCs (×10^6^)/μl	4.33 ± 0.065	41.80 ± 2.20^a^	32.90 ± 1.65^c^	38.71 ± 1.92^ab^	35.64 ± 0.85^bc^	39.66 ± 1.51^ab^	23.79 ± 1.62^d^	32.74 ± 1.88^c^
WBCs (×10^3^) /μl	7.78 ± 0.012	**50.53 ± 0.87**^**b**^	**53.54 ± 0.33**^**a**^	**52.14 ± %0.49**^**a**^	**50.39 ± 0.35**^**b**^	**48.46 ± 0.15**^**c**^	**45.67 ± 0.57**^**d**^	**45.28 ± 0.46**^**d**^
Platelets (×10^3^)/μl	344 ± 3.19	44.08 ± 0.79^e^	56.42 ± 0.58^b^	61.81 ± 0.75^a^	52.37 ± 0.89^c^	58.15 ± 0.76^b^	49.76 ± 0.58^d^	45.25 ± 0.86^e^
Endogenous antioxidant enzymes								
CAT (U/min)	0.5 ± 0.04	342.67 ± 8.33^b^	84.67 ± 15.53^d^	100 ± 1.20^d^	274 ± 12.49^c^	285.3 ± 27.15^c^	315.3 ± 17.47^bc^	410 ± 30.27^a^
POD (U/min)	1 ± 0.04	209 ± 6.56^b^	70.33 ± 2.52^e^	122 ± 1.73^d^	63.33 ± 4.16^e^	317.67 ± 3.21^a^	183.67 ± 9.07^c^	301 ± 10.15^a^
SOD (U/mg protein)	0.96 ± 0.05	261.81 ± 3.66^a^	154.17 ± 2.08^e^	198.96 ± 4.17^c^	190.63 ± 2.08^cd^	232.29 ± 3.76^b^	176.7 ± 10.43^d^	191.6 ± 10.26^cd^
GST (nM/min/ml)	81.01 ± 0.44	191 ± 1.81^b^	33.72 ± 0.78^e^	99.22 ± 0.87^c^	34.43 ± 1.28^e^	266.29 ± 1.98^a^	67.07 ± 0.78^d^	101.08 ± 1.91^c^
Biochemical parameters								
ALT (U/L)	93.20 ± 2.64	**50.67 ± 2.01**^**b**^	**50.83 ± 2.45**^**b**^	**54.36 ± 1.92**^**ab**^	**52.47 ± 1.78**^**ab**^	**56.96 ± 2.43**^**a**^	**58.13 ± 2.10**^**a**^	**57.46 ± 1.70**^**a**^
AST (U/L)	67.80 ± 2.05	**59.73 ± 0.94**^**bc**^	**65.23 ± 1.50**^**a**^	**61.91 ± 1.27**^**ab**^	**54.72 ± 1.89**^**de**^	**58.58 ± 1.39b**^**cd**^	**56.96 ± 1.01**^**cde**^	**53.79 ± 1.69**^**e**^
ALP (U/L)	356 ± 5.69	**60.69 ± 1.01**^**bcd**^	**58.38 ± 0.92**^**de**^	**61.14 ± 1.14**^**abc**^	**57.28 ± 0.81**^**e**^	**59.43 ± 0.35**^**cde**^	**62.15 ± 1.01**^**ab**^	**63.38 ± 1.13**^**a**^
Urea (mg/dl)	72.2 ± 2.76	**62.59 ± 1.41**^**a**^	**51.01 ± 1.37**^**c**^	**57.02 ± 2.44**^**ab**^	**50.15 ± 1.18**^**c**^	**52.29 ± 1.71**^**bc**^	**61.52 ± 2.84**^**a**^	**59.65 ± 2.60**^**a**^
Creatinine (mg/dl)	2.89 ± 0.45	**61.01 ± 5.04**^**a**^	**60.21 ± 3.34**^**a**^	**61.82 ± 4.19**^**a**^	**60.32 ± 3.89**^**a**^	**62.40 ± 2.78**^**a**^	**61.25 ± 3.51**^**a**^	**61.94 ± 2.50**^**a**^
Bilirubin (mg/ml)	7.56 ± 0.97	**65.21 ± 2.82**^**a**^	**58.38 ± 1.23**^**a**^	**63.10 ± 8.54**^**a**^	**59.79 ± 4.37**^**a**^	**60.76 ± 3.44**^**a**^	**68.17 ± 1.07**^**a**^	**68.08 ± 5.16**^**a**^
Total proteins (g/dl)	2.14 ± 0.11	214.02 ± 18.19^a^	185.51 ± 23.36^a^	224.45 ± 27.63^a^	213.55 ± 29.55^a^	210.28 ± 23.22^a^	186.29 ± 17.50^a^	195.02 ± 19.61^a^
Lipid peroxidation assay								
TBARs (nM/min/mg protein)	133.7 ± 2.61	**35.74 ± 0.82**^**a**^	**15.08 ± 1.65**^**d**^	**24.27 ± 1.11**^**c**^	**15.12 ± 1.74**^**d**^	**21.39 ± 1.22**^**c**^	**37.41 ± 0.89**^**a**^	**31.01 ± 1.32**^**b**^

Results of disease control are expressed as mean ± SD (*n* = 6, where *n* = number of rats analysed in each group). Alleviative effects of remaining groups are expressed as percent increase or decrease (**bold**) of tested parameters in comparison to the disease control group. Groups are compared statistically to the positive control group and superscripts (a-e) in individual rows specifying significance at *p < 0.05*

## Discussion

Irregularities in the molecular processes within the cell if not alleviated and mitigated promptly may ultimately result in tumor cell proliferation. Natural remedies are long considered legitimate starting materials for discovery of novel anticancer agents. Since traditional consumption of plants as herbs and medicine have been practiced for many centuries, we have chosen two *Datura* species and designed our study to evaluate their anticancer potential by employing a wide spectrum of in vitro anticancer assays. Moreover, in vivo antileukemic assay of potent extracts was also performed. A schematic plan was followed in execution of current study. Preliminary phytochemical screening and in vitro assessment of the antioxidant potential of 32 (16 for each specie) extracts of root, stem, leaf and flower parts of selected *Datura* species was the starting point of this study. It was followed by selection of most active extracts based on observed results. After toxicity studies of chosen extracts, they were utilized in predetermined doses for the estimation of in vivo antileukemic activity in rats.

Ethyl acetate leaf extracts of *Datura stramonium* (DSL-EA) and *Datura inoxia* (DIL-EA) were the two extracts selected out of 32 owing to their efficacy in preliminary testing. Phytochemical screening of DSL-EA and DIL-EA showed TPC of 28.67 ± 0.97 and 27.69 ± 1.12 μg GAE/mg extract, while TFC of 16.16 ± 0.06 and 20.17 ± 0.17 μg QE/mg extract respectively. Polyphenolic compounds i.e. flavonoids, tannins and phenolic acids are the chief contributor to antioxidant properties of majority of medicinal plants. The presence of important functional groups like ketonic, hydroxyl, phenolic and methoxy render these compounds significant antioxidant activity [32, 33]. These compounds are considered as pharmacologically important due to properties like inhibition of lipid peroxidation and free radical scavenging [19, 20]. HPLC-DAD quantification revealed notable quantities of important polyphenols including rutin, gallic acid, catechin and apigenin in DSL-EA and caffeic acid in DIL-EA. Detection of all these polyphenols further potentiates the medicinal value of selected *Datura* species. The polyphenols detected and quantified in selected extracts have established pharmacological uses i.e. rutin is known for its anticancer, antidiabetic and antioxidant properties [34], apigenin has known anticancer and anti-inflammatory potential [35] and it is also known to induce autophagy in leukemia cells [19]. Gallic acid has numerous medicinal uses particularly its application as antiangiogenic, anticancer, anti-inflammatory and as an antimicrobial agent [36]. Antioxidant properties of caffeic acid are extensively studied, in one such study it surpassed numerous other antioxidants in curbing aflatoxin generation by more than 95% [7]. Apart from antioxidant properties, these polyphenols have documented likelihood to impede cancer cell proliferation, activation of pro-carcinogens and inhibit growth signaling pathways and thus prevent cancer cell proliferation [37, 38]. A study has reported substantial correlation between the occurrence of polyphenols and in vivo tumor growth inhibition via regulating MAPK/ERK, PI3K/AKT and STAT3 pathways in lung metastasis and breast cancer stem cells [39]. The antioxidant potential (TAC and TRP) displayed by DSL-EA (160.92 ± 3.00 and 50.43 ± 1.72 μg AAE/mg extract respectively) and DIL-EA (140.44 ± 5.6 and 44.02 ± 1.30 μg AAE/mg extract respectively) might well be attributed to the presence of these polyphenols along with many others which are not quantified in the present study.

Cytotoxicity testing of extracts using a series of in vitro models including brine shrimps (*Artemia salina)* larvae*, Streptomyces* 85E strain and activity against different cancer cell lines was also performed. Bioactivity profile was established initially by testing the extracts against brine shrimps larvae. Results showed a concentration dependent lethality of DSL-EA and DIL-EA against shrimps. The determination of cytotoxic potential of natural products can be determined reliably with the aid of brine shrimp lethality assay [40]. This assay is typically performed to deduce the safety and quantify the bioactivity of plant extracts using a simple zoological organism. Plant extracts with LC_50_ values less than 1000 μg/ml against brine shrimps larvae are considered to possess cytotoxic potential [41]. Extremely significant results necessitated further scrutiny using available models to validate the cytotoxicity of extracts to reinforce our findings.

Extracts were also screened for their protein kinase inhibitory potential. *Streptomyces* 85E require protein kinases for their aerial hyphae formation which is the basic principle behind the performance of this assay. Kinases play a key role in cell proliferation, division and are considered as critical factors in carcinogenesis [42, 43]. This assay is thus performed for the determination of kinase inhibitory and ultimately anticancer action of extracts. Bald phenotypic zones of 12.5 mm (MIC: 100 ± 3.03 μg/disc) and 19 mm (MIC: 25 ± 1.62 μg/disc) were displayed by DSL-EA and DIL-EA respectively which confirm substantial protein kinase inhibitory activity. Recently, the development of protein kinase inhibitors from natural sources particularly from plants has generated renewed interest in targeting kinases. It is because of the fact that protein kinases have a major role to play in cell growth and development, progression of cell cycle and signals transduction across the nuclear membrane [20]. Mutations in genes specifically important for cell growth, differentiation and death result in elevated kinase activity at serine/threonine residues usually found in human cancers [44]. Serine/threonine kinases play a critical role in carcinogenesis [42]. Likewise, a study has shown alteration in a subfamily of serine/threonine kinases in ovarian carcinomas [45]. Tyrosin kinases are involved in regulation of cellular functions of normal cells, additionally they play a key role in oncogenesis [43]. It is for this reason that kinase inhibitors have emerged as auspicious targets for cancer treatment [46]. A definite advantage of using whole cell *Streptomyces* is that the cytotoxic activity of samples can be readily identified using this assay. Furthermore, inhibitors of signal transduction for numerous applications i.e. antitumor, anti-infective and anti-mycobacteria can also be identified with the aid of this simple assay [47].

Anticancer potential of *Datura* extracts was further assessed against selected cancer cell lines. PC-3 is a human prostate cancer cell line and is used in exploration of numerous biochemical variations in prostate cancer cells and assessment of their response to different anticancer agents. Prostate cancer is one of the most widespread cancers diagnosed in men and developing new chemotherapeutic agents for its treatment has been quite a challenge [48]. Human breast cancer cell lines MDA-MB 231 and MCF-7 were also used in the current study. Breast cancer is a major health problem in women, one in ten of all reported cancers annually is a cancer of female breast [9]. Out of the two *Datura* species studied, DIL-EA exhibited remarkable activity against each of the three tested cancer cell lines with IC_50_ values lower than 3.00 μg/ml in each case. DSL-EA on the contrary displayed comparatively weak cytotoxic activity against the chosen cancer cell lines. The anticancer potential of aerial parts and seeds of *Datura* species against cancer cell lines has been explored previously by numerous researchers using different solvent extracts. DS methanol extract of seeds when tested against MCF-7 cell line displayed an IC_50_ value of 113.05 μg/ml and the observed activity was postulated to be due to the detected antioxidant potential of the same extract [18]. In another study DS water extract of leaf part was tested at a concentration of 1 mg/ml against numerous cell lines including MDA-MB 231 and 24 h exposure led to a significant decline in cell survival rate [49]. DI methanloic leaf extract and one of its constituents from the extract named ‘Dinoxin B’ were tested for anti-proliferative action against a range of normal and human cancer cell lines. Sub-micromolar IC_50_ values were observed against different cancer cells and the most sensitive ones were various breast cancer cell lines. The activity was accredited to the presence of Dinoxin B found specifically in leaf portion of *D.inoxia* [14]. Dinoxin B is fom the withanolide class of natural products. Keeping many of the previously reported studies in sight, we can infer that antioxidant moieties as well as different withanolides reported in *Datura* species might well be responsible for the remarkable anticancer activity of DIL-EA as well as the modest activity profile of DSL-EA.

Lymphocyte toxicity assessment revealed no significant cytotoxic action of either DSL-EA or DIL-EA against normal human lymphocytes. Acquired results affirmed the selective nature of cytotoxic action of *Datura* extracts against cancer cells. Results of acute toxicity study further supported the plan to perform in vivo antileukemic assay as both species of *Datura* at doses up to 2 g/kg were found to have no damaging and deleterious effects on rats used in the study.

**Fig. 5:**
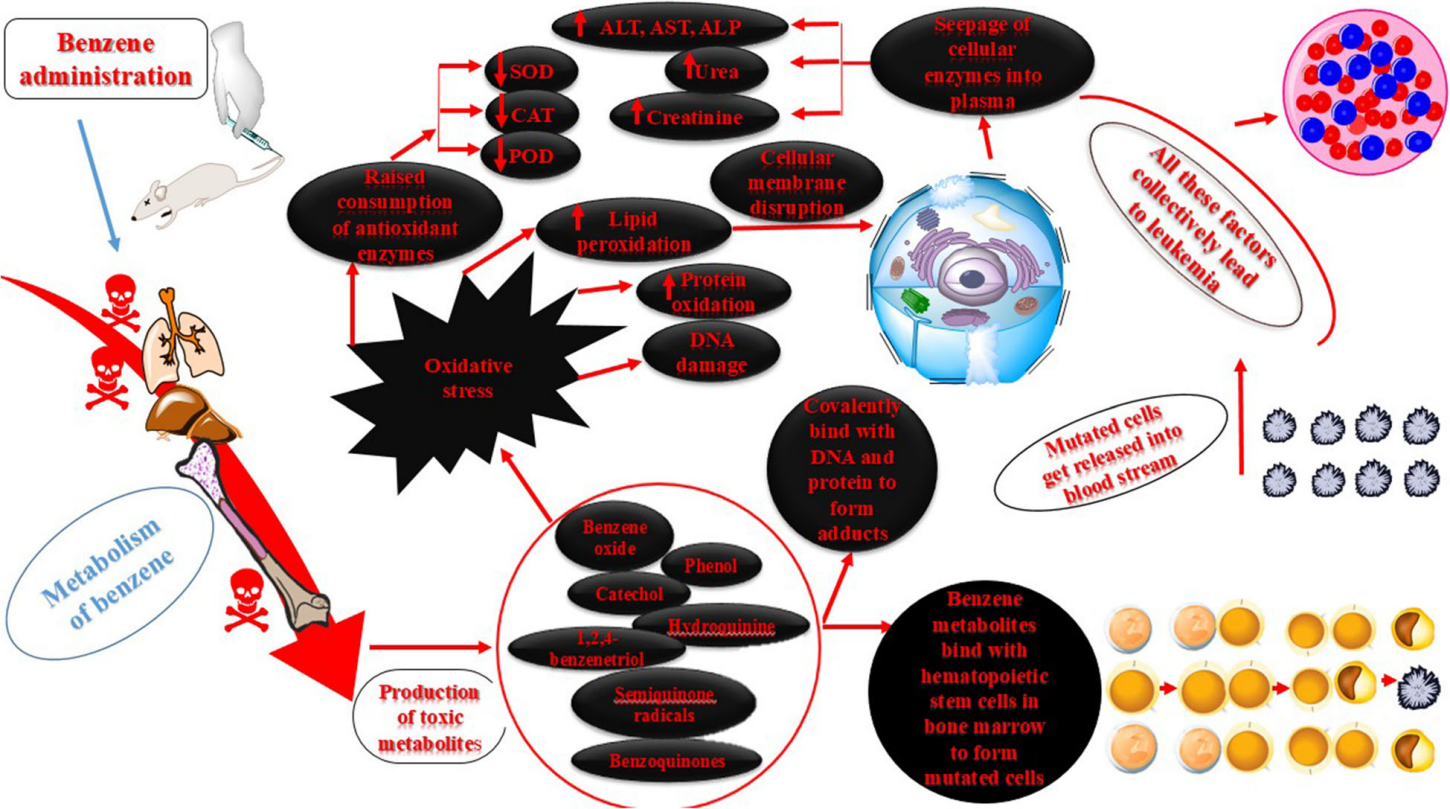
Proposed mechanism of Benzene induced toxicity leading to leukemia. Blue and white colour represent normal functioning within rat body while black and red colour represents the benzene induced distortions. The figure is a simplified version of possible mode of benzene induced neoplasia and crosstalk between different pathways has been omitted. CAT-Catalase, POD-Peroxidase, SOD-Superoxide dismutase, ALT-Alanine transaminase, ALP- Alkaline phosphatase, AST- Aspartate transaminase [56–58].. Clip art images used in the figure were made using ChemDraw Professional v19.0

Leukemia is amongst the most prominent cancer forms both in infants and adults and is characterized by unrestrained formation, multiplication and accumulation of malignant white blood cells in bone marrow and peripheral blood [50]. The exact cause of leukemia is fundamentally unknown but occupational and environmental factors are considered as significant contributors to the onset and progression of the disease. Mutations in DNA as a result of oncogenes activation or deactivation of tumor suppression genes is considered the main causative factor of leukemia. Apart from sporadic mutations, few occur as a result of certain predisposing factors like exposure to different carcinogens [51]. One such carcinogen is benzene which is associated with many health hazards. The induction of hematological toxicity by benzene has been reported over a century ago and fresh studies have also linked benzene to haematotoxicity even at concentrations less than 1 ppm in air [52, 53]. Benzene cause acute myeloid leukemia (AML) and myelodysplastic syndrome and is also a plausible cause of other hematological disorders including non-Hodgkin lymphoma [54, 55]. The postulated mode of action of benzene (Fig. 5**)** briefly consists of five major steps: (a) benzene metabolism to its metabolite, benzene oxide; (b) its interaction with target cells in the bone marrow; (c) initiation of mutations in bone marrow cells; (d) designated clonal proliferation of these mutated cells and (e) abnormal growth and multiplication of target cells in bone marrow resulting in neoplasia (leukemia) [56].

The antileukemic potential of DSL-EA and DIL-EA on male Sprague Dawley rats bearing benzene induced leukemia was assessed on the basis of results acquired from a series of haematological, biochemical, enzymatic and histological studies. Significantly decreased RBCs, platelets and haemoglobin levels while elevated WBCs and ESR in disease control rats established the fact that leukemia was induced in this group. Marked elevation in WBCs count is one of the prominent signs of acute leukemic condition. Results revealed a 40–50% decrease in WBCs count in study groups when compared to negative control. Leukemia induction in rats by benzene administration is previously reported in numerous studies [11, 26, 27, 59]. Comparison of study groups clearly revealed the protective effect of low and high doses of *Datura* extracts. There was no statistically significant difference (*p* < 0.05) between the calculated haematological parameters of treatment and preventive groups when compared with the positive control group. It showed that administered doses of extracts have shown fairly comparable antileukemic activity to the standard drug used in current study. Polyphenols detected in phytochemical investigation might be responsible for potentiating the vasoprotective and vasotonic effects of DSL-EA and DIL-EA.

Exposure to benzene has been reported to cause liver damage since it is the major organ responsible for compound metabolism into inert and potentially toxic metabolites. Enzymatic conversion of benzene to reactive species can lead to oxidative stress. These species can interact with tissues and cell membranes causing lipid peroxidation which can further aggravate liver injury [60–62]. Abnormalities in level of serum enzymes and macromolecules signifying liver functionality, including ALT, AST and ALP as well as bilirubin and total proteins clearly indicated liver injury in disease control rats. The incidence of liver injury is very common in hematological diseases particularly leukemia [63]. Preventive and treatment groups of both extracts reverted liver damage to a great extent as evident by their statistically significant (p < 0.05) difference from disease control group. Although the biochemical mechanism for regeneration of liver enzymes and functionality is not clear, nevertheless inhibition of lipid peroxidation and free radical scavenging properties of polyphenolic compounds might be responsible for curbing the damage caused to liver by benzene [19, 20] .

Kidney acts as one of the major organs responsible for detoxification and waste elimination rendering it highly susceptible to toxins induced injury. Benzene induced nephrotoxicity has been documented previously [62]. Glomerulonephritis and nephrotic syndrome without evident kidney failure have been reported in leukemia and lymphoma. Tumor lysis and cell bursting in acute leukemic conditions result in elevated potassium, phosphate and decreased calcium level along with acid-base imbalance. All these anomalies result in kidney failure characterized initially by significantly elevated serum urea and creatinine level [64]. Benzene administration posed significant nephrotoxic effects. Disease control rats were the most affected ones based on highest estimated values of selected nephrotoxicity markers. *Datura* species at low and high doses lowered urea and creatinine levels in a manner comparable to the positive control. Nephroprotective effect observed in the current study further strengthened the potential antileukemic action of used extracts.

The role played by oxidants in different stages of carcinogenesis is well documented [65]. Cells using aerobic metabolism for their energy needs, inevitably generate ROS [66]. Extensive accumulation of ROS mediate harm to biomolecules and causes deleterious effects resulting in numerous diseases including haematopoietic malignancies [67]. There is a well-documented association between oxidative stress and leukemia, higher amounts of ROS are generated by leukemic cells as compared to normal cells because they are under a recurrent state of oxidative blockade [68]. The damaging effects mediated by ROS can be nullified by action of enzymatic and non-enzymatic antioxidants. Endogenous antioxidant enzymes including CAT, POD, SOD and GST play a significant role in combating oxidative stress [67]. Conversion of H_2_O_2_ to H_2_O and O_2_ via catalytic conversion is triggered by CAT, which is a major antioxidant enzyme present in aerobic cells. SOD, on the other hand dismutates superoxide anion radical (2O_2_) to less toxic H_2_O_2_ and molecular oxygen/O_2_ by redox mechanism. A selenoprotein prevalent in cytosol and mitochondrial matrix, POD, is involved in the catalytic reduction of lipid peroxides and H_2_O_2_ [30]. GSTs are evolutionarily conserved enzymes that play a key role in detoxification of numerous xenobiotics. The conjugation of reduced glutathione (GSH) to electrophilic substrates is catalyzed by GSTs resulting in production of easily soluble and less toxic compounds [69]. Level of these endogenous enzymes is disturbed due to oxidative overload. There are contradictory reports in the literature regarding the level of endogenous antioxidant enzymes in various types of cancers [58]. In our study, benzene treated disease control group showed severely reduced activity levels of CAT, POD, SOD and GST. These findings reinforce the observed aberrations perpetrated by the leukemogenic chemical used in current study. Excessive ROS generation and extended oxidative stress following benzene doses and leukemia induction might have led to exhaustion of endogenous antioxidant defense system particularly the antioxidant enzymes. Furthermore, the expression of endogenous antioxidant enzymes might also be adversely affected. Administration of EA leaf extracts of both plants ameliorated the oxidative stress in a dose dependent manner. Low doses yielded mild restorative effects while greater results were observed in case of high doses of both preventive and treatment modes. The restorative effect might well be accredited to the presence of useful polyphenols and other antioxidant moieties within *Datura* species.

Weakened antioxidant defense system result in increased lipid peroxidation with consequent deterioration in cellular membrane integrity and cellular functions. There are numerous end products of lipid peroxidation including TBARs. These are considered as effective markers of oxidative disorders and numerous cancers including leukemia [27, 70]. As revealed by the estimated biochemical parameters, the antioxidant defense system of disease control rats was highly affected. Inflated oxidative degradation of lipids was thus an anticipated outcome. Both extracts expressively lowered TBARs level in treatment mode, while high doses in preventive mode also resulted in mild recovery. Escalated NO level in disease control rats further press the possibility of an irreversible damage to lipoproteins and cellular membrane owing to the tendency of NO to react with **·**O_2_ and generation of highly volatile peroxynitrite. These aberrations were also curbed by high and low doses of extracts in identical manner.

Detection of medicinally important phytochemicals, endowed with antioxidant activity in current study and the documented evidence of occurrence of compounds i.e. withanloides [71] may be responsible for the observed anticancer action of *Datura* extracts. Withanolides have proven cytotoxic action in numerous cancer cell types including leukemia. These compounds act through diverse molecular mechanisms i.e. induction of apoptosis via down regulation of akt phosphorylation [72], or through activation of p38 mitogen activated protein kinase (MAPK) signaling cascade resulting in elevated levels of BAX (Bcl-2- associated X protein) and ultimately, initiation of mitochondrial cell death [73]. Findings of our study further deduce the alleviating effects of selected species of *Datura* in benzene induced leukemia. Despite the fact that animal models have numerous common properties with human physiology, due diligence must be performed when trying to extrapolate findings from an animal model of a disease to a clinical trial setting.

## Conclusion

In view of the results obtained in phytochemical and biological evaluation of different extracts of *D. stramonium* and *D. inoxia*, a comprehensive study targeting the anticancer prospects of selected extracts seemed highly prudent. The presence of important secondary metabolites including phenolics and flavonoids with substantial antioxidant properties further support the undertaken study. Significant in vitro anticancer activity in addition to relatively selective action against cancerous cells deemed these extracts as suitable candidates for subsequent in vivo evaluation. Antileukemic action as appraised by haematological, biochemical and histological evaluation as well as endogenous antioxidant enzymes levels clearly indicate the significant ameliorating effects of selected extracts against benzene induced leukemia. Both species yielded comparable brine shrimp lethality, protein kinase inhibitory and in vivo antileukemic results while *D.inoxia* showed significantly greater in vitro anticancer potential against prostate and breast cancer cell lines. The findings from the current study fully support the need for detailed molecular investigation to determine the mechanism(s) responsible for the observed anticancer actions.

## Supplementary information

**Additional file 1.** Fig Ad2a. Chromatogram of standard polyphenols.

**Additional file 2.** Fig Ad2b. Chromatogram of compounds detected in DSLEA

**Additional file 3 **Fig Ad2c. Chromatogram of compounds detected in DILEA*.*

**Additional file 4.** Fig Ad3a. Histopathological observations (10X) of Gp I liver.

**Additional file 5.** Fig Ad3b. Histopathological observations (10X) of Gp 2 liver.

**Additional file 6.** Fig Ad3c. Histopathological observations (10X) of Gp 3 liver.

**Additional file 7.** Fig Ad3d. Histopathological observations (10X) of Gp 4 liver.

**Additional file 8.** Fig Ad3e. Histopathological observations (10X) of Gp 5 liver.

**Additional file 9.** Fig Ad3f. Histopathological observations (10X) of Gp 6 liver.

**Additional file 10.** Fig Ad3g. Histopathological observations (10X) of Gp 7 liver.

**Additional file 11.** Fig Ad3h. Histopathological observations (10X) of Gp 8 liver.

**Additional file 12.** Fig Ad3i. Histopathological observations (10X) of Gp 9 liver.

**Additional file 13.** Fig Ad4a. Histopathological observations (10X) of Gp 1 Kidney.

**Additional file 14.** Fig Ad4b. Histopathological observations (10X) of Gp 2 Kidney.

**Additional file 15.** Fig Ad4c. Histopathological observations (10X) of Gp 3 Kidney.

**Additional file 16.** Fig Ad4d. Histopathological observations (10X) of Gp 4 Kidney.

**Additional file 17.** Fig Ad4e. Histopathological observations (10X) of Gp 5 Kidney.

**Additional file 18.** Fig Ad4f. Histopathological observations (10X) of Gp 6 Kidney.

**Additional file 19.** Fig Ad4g. Histopathological observations (10X) of Gp 7 Kidney.

**Additional file 20.** Fig Ad4h. Histopathological observations (10X) of Gp 8 Kidney.

**Additional file 21.** Fig Ad4i. Histopathological observations (10X) of Gp 9 Kidney.

## Data Availability

The datasets used and/or analyzed during the current study are available from the corresponding author on reasonable request.

## Abbreviations

BAX: Bcl-2- associated X protein
DCV: Damaged central vein
DIL-EA: *Datura inoxia* leaf extract in ethyl acetate
DSL-EA: *Datura stramonium* leaf extract in ethyl acetate
RP-HPLC: Reverse phase high performance liquid chromatography
ALT: Alanine transaminase
ALP: Alkaline phosphatase
AST: Aspartate transaminase
DMSO: Dimethyl sulfoxide
AAE: Ascorbic acid equivalent
GAE: Gallic acid equivalent
QE: Quercetin equivalent
TSB: Trypron soya broth
PBS: Phosphate buffer saline
HIFBS: Heat inactivated foetal bovine serum
MIC: Minimum inhibitory concentration
OECD: Organization for Economic Cooperation and Development
CPK: Creatinine phosphokinase
GST: Glutathione S-transferase
SOD: Superoxide dismutase
CAT: Catalase
MAPK: Mitogen activated protein kinase
POD: Peroxidase.
TBARS: Thiobarbituric acid reactive substances.
ESR: Erythrocyte sedimentation rate.
TPC: Total phenolics content.
TFC: Total flavonoids content.
TAC: Total antioxidant capacity.
TRP: Total reduction potential.
DPPH: 2-2-diphenyl 1-picrylhydrazyl
