# Supplementary figures and images for: Preclinical anticancer studies on the ethyl acetate leaf extracts of Datura stramonium and Datura inoxia
Source: BMC Complement Med Ther. 2020 Jun 17;20:188. doi: 10.1186/s12906-020-02975-8 (PMC7302377; doi:10.1186/s12906-020-02975-8)

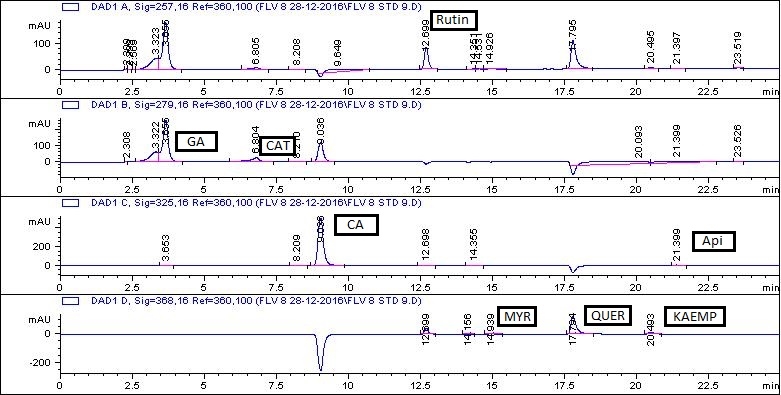

Supplement: Supplementary file 1 — Additional file 1. Fig Ad2a. Chromatogram of standard polyphenols. [file 12906_2020_2975_MOESM1_ESM.jpg]

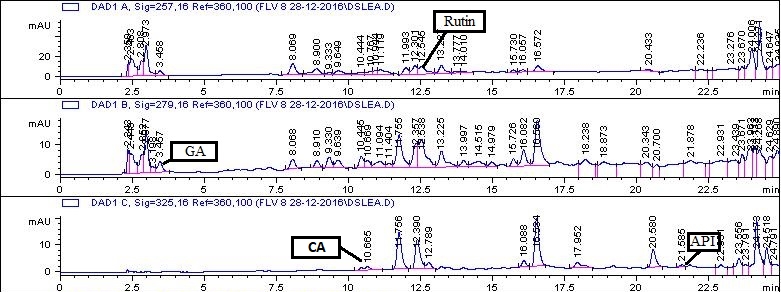

Supplement: Supplementary file 2 — Additional file 2. Fig Ad2b. Chromatogram of compounds detected in DSLEA [file 12906_2020_2975_MOESM2_ESM.jpg]

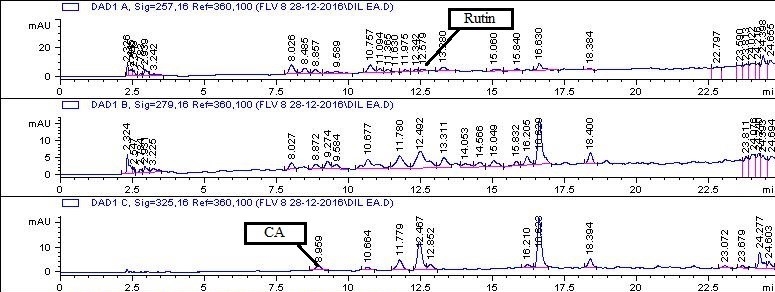

Supplement: Supplementary file 3 — Additional file 3 Fig Ad2c. Chromatogram of compounds detected in DILEA. [file 12906_2020_2975_MOESM3_ESM.jpg]

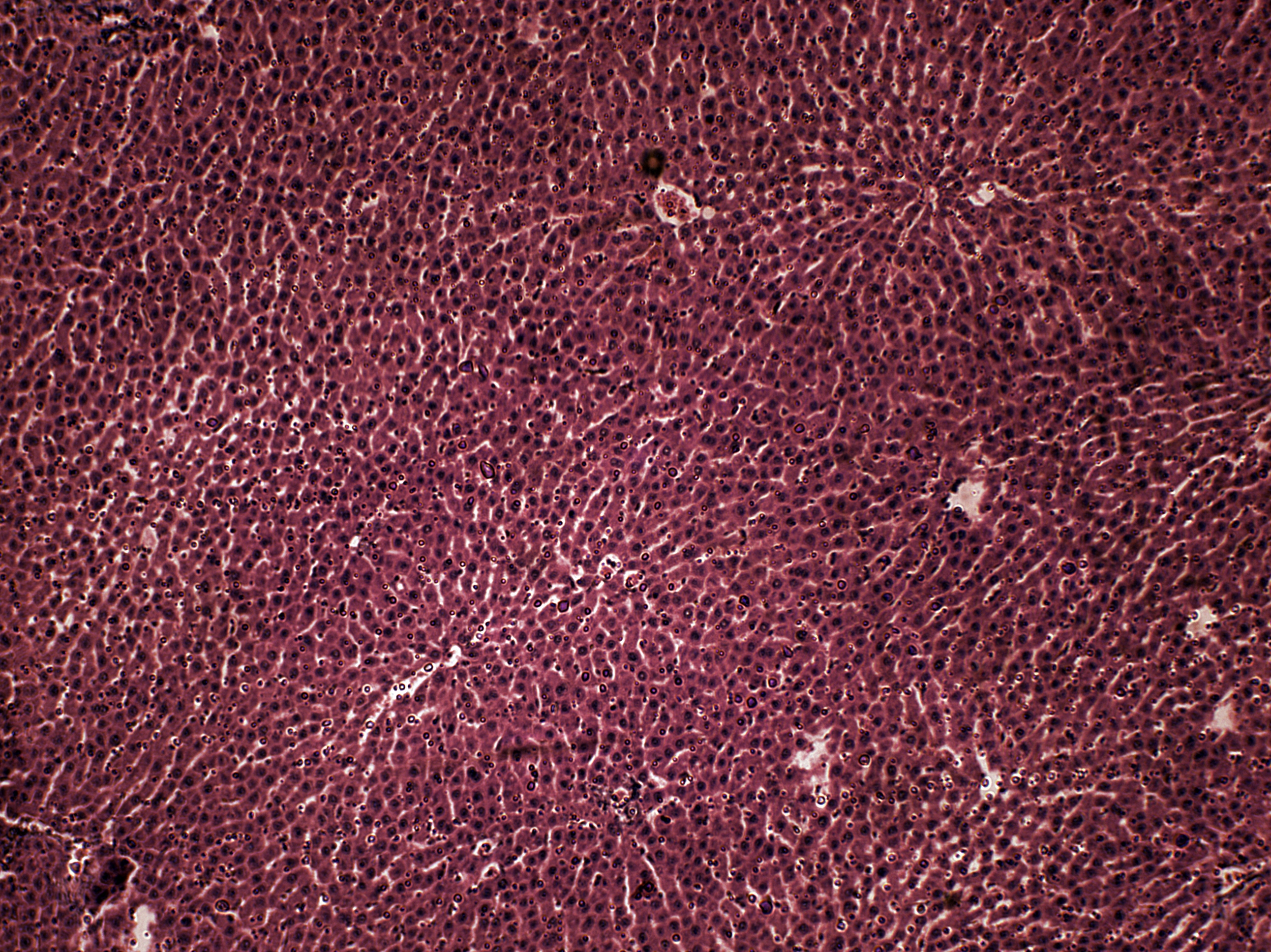

Supplement: Supplementary file 4 — Additional file 4. Fig Ad3a. Histopathological observations (10X) of Gp I liver. [file 12906_2020_2975_MOESM4_ESM.jpg]

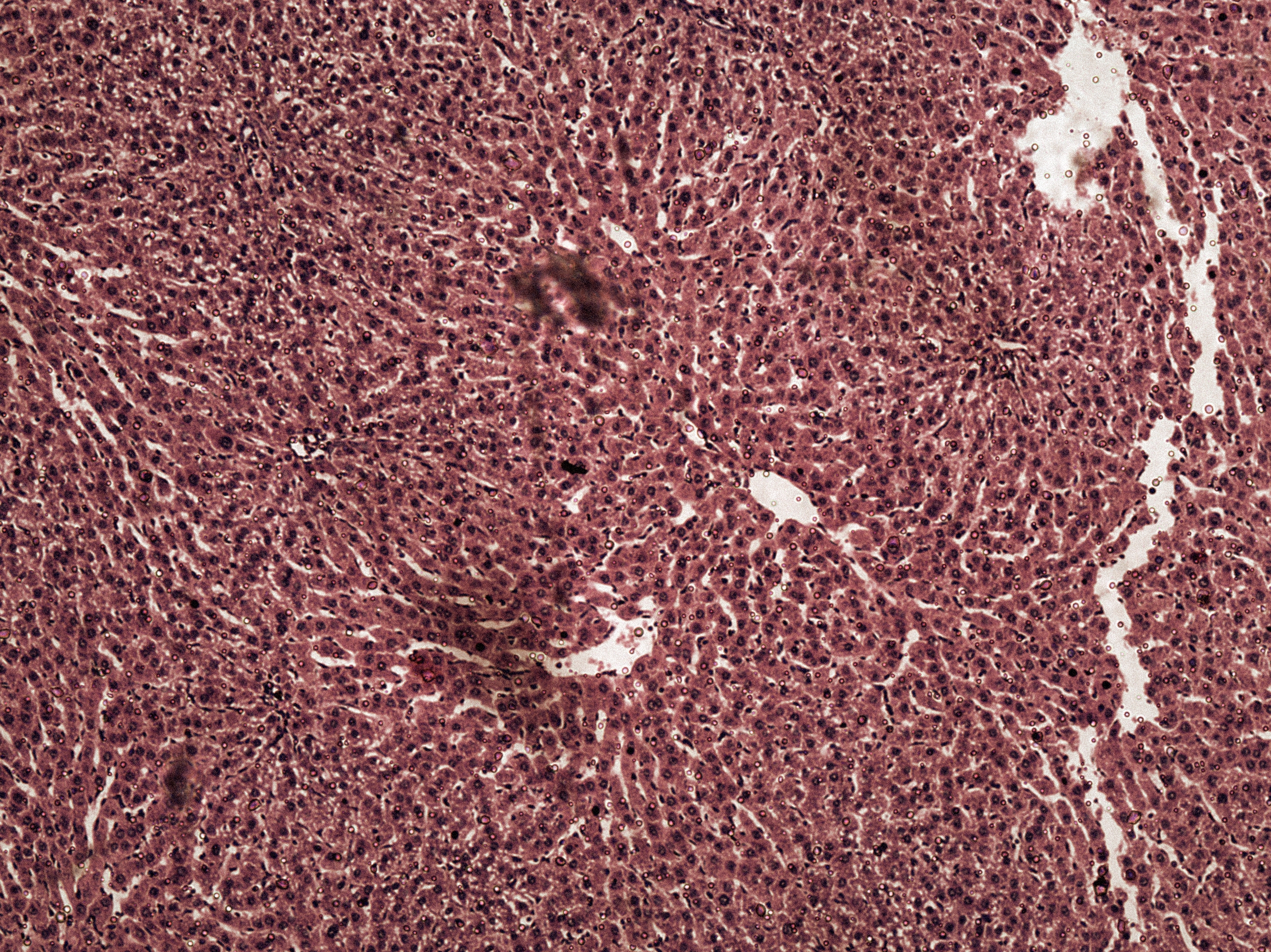

Supplement: Supplementary file 5 — Additional file 5. Fig Ad3b. Histopathological observations (10X) of Gp 2 liver. [file 12906_2020_2975_MOESM5_ESM.jpg]

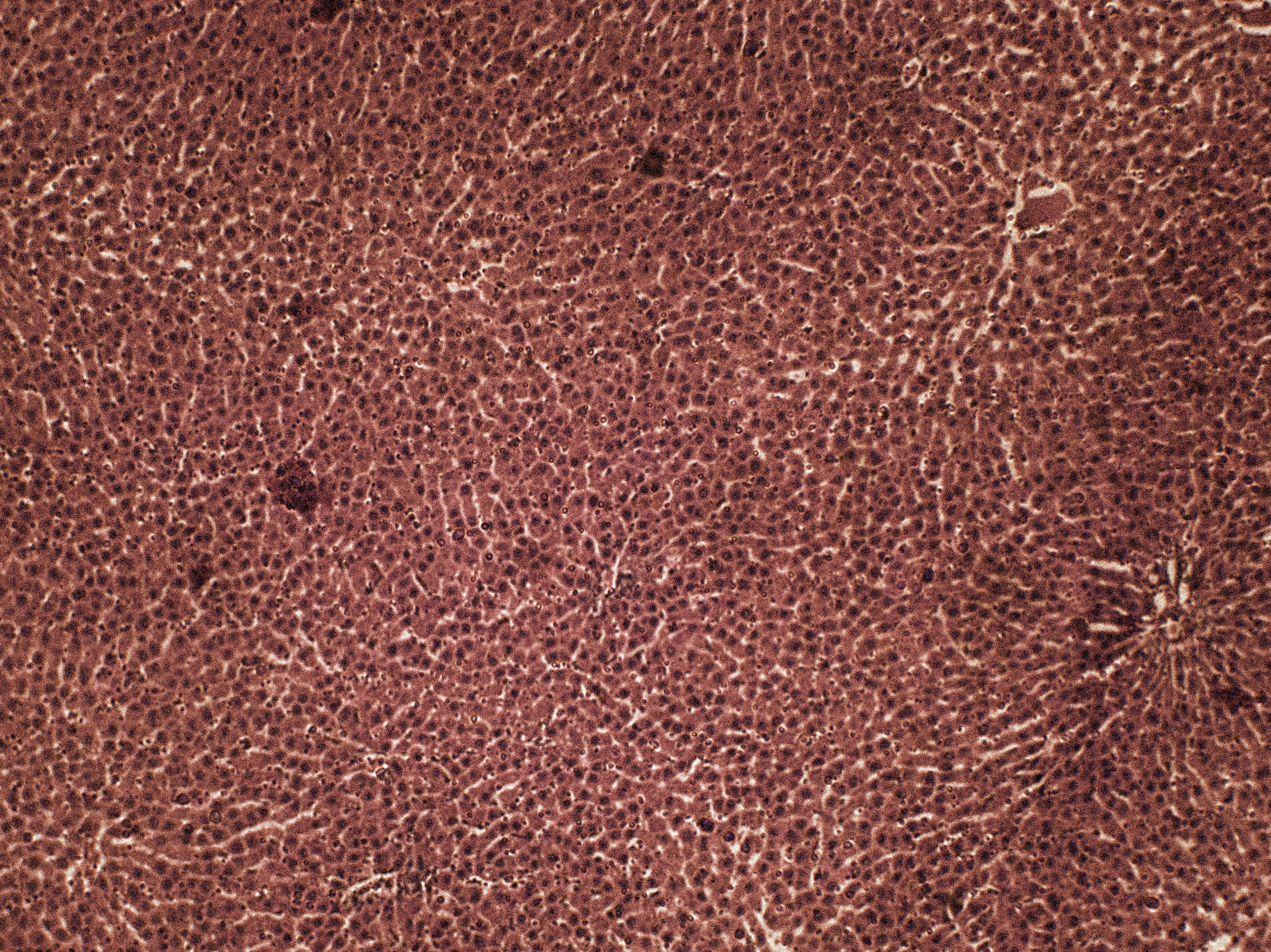

Supplement: Supplementary file 6 — Additional file 6. Fig Ad3c. Histopathological observations (10X) of Gp 3 liver. [file 12906_2020_2975_MOESM6_ESM.jpg]

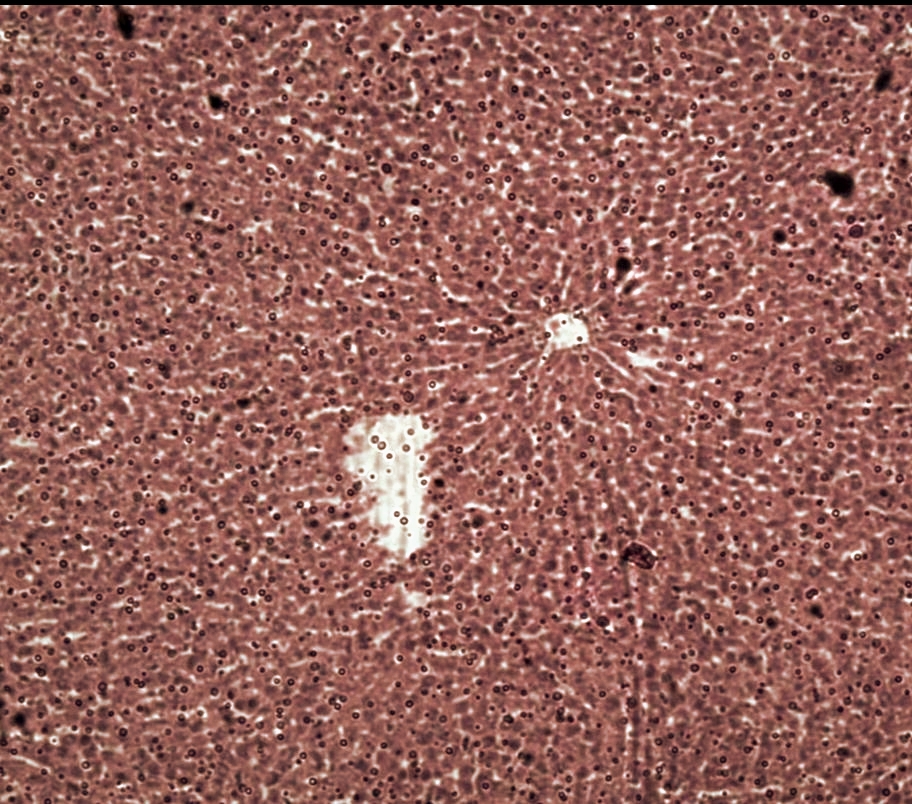

Supplement: Supplementary file 7 — Additional file 7. Fig Ad3d. Histopathological observations (10X) of Gp 4 liver. [file 12906_2020_2975_MOESM7_ESM.jpg]

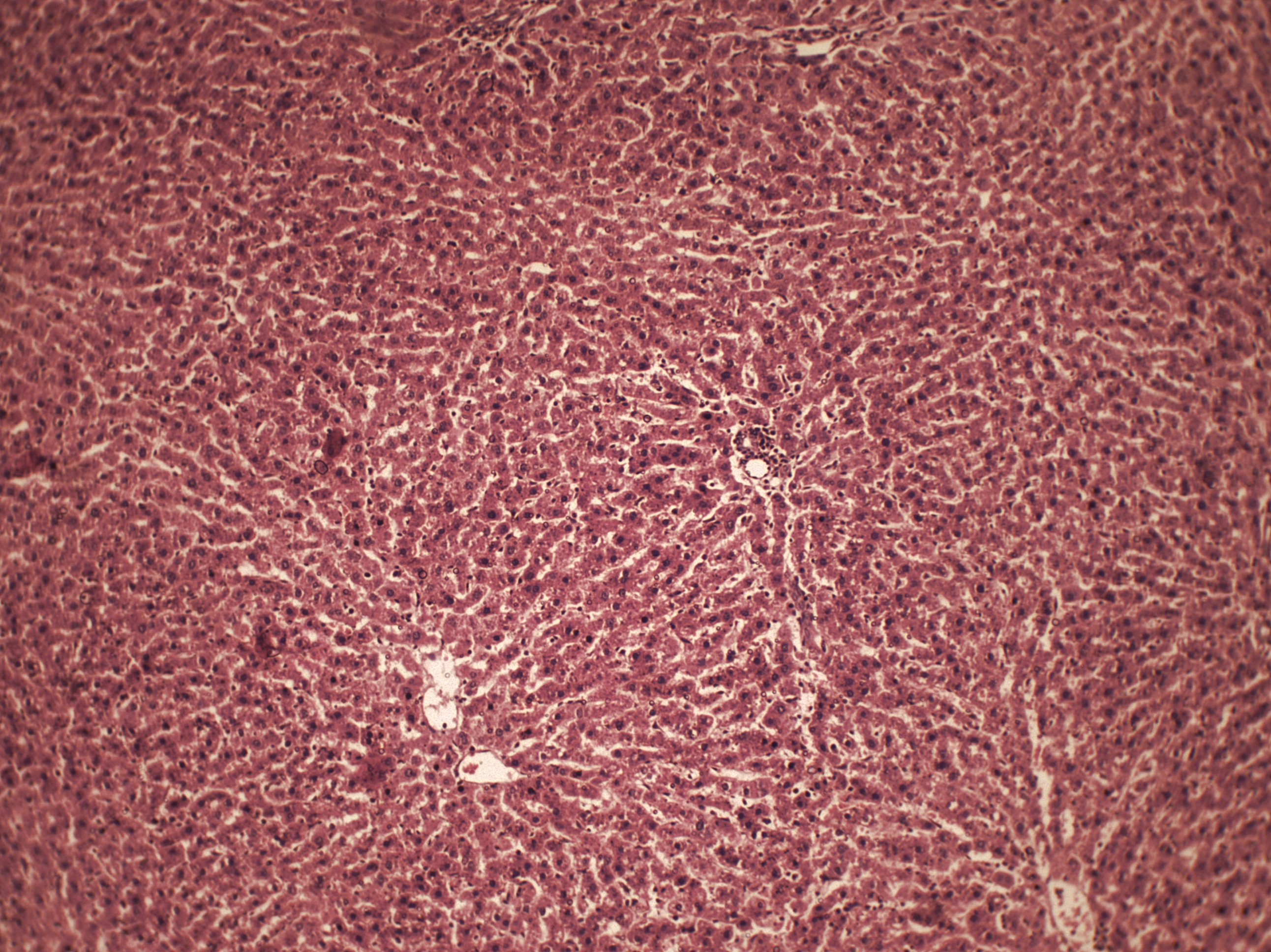

Supplement: Supplementary file 8 — Additional file 8. Fig Ad3e. Histopathological observations (10X) of Gp 5 liver. [file 12906_2020_2975_MOESM8_ESM.jpg]

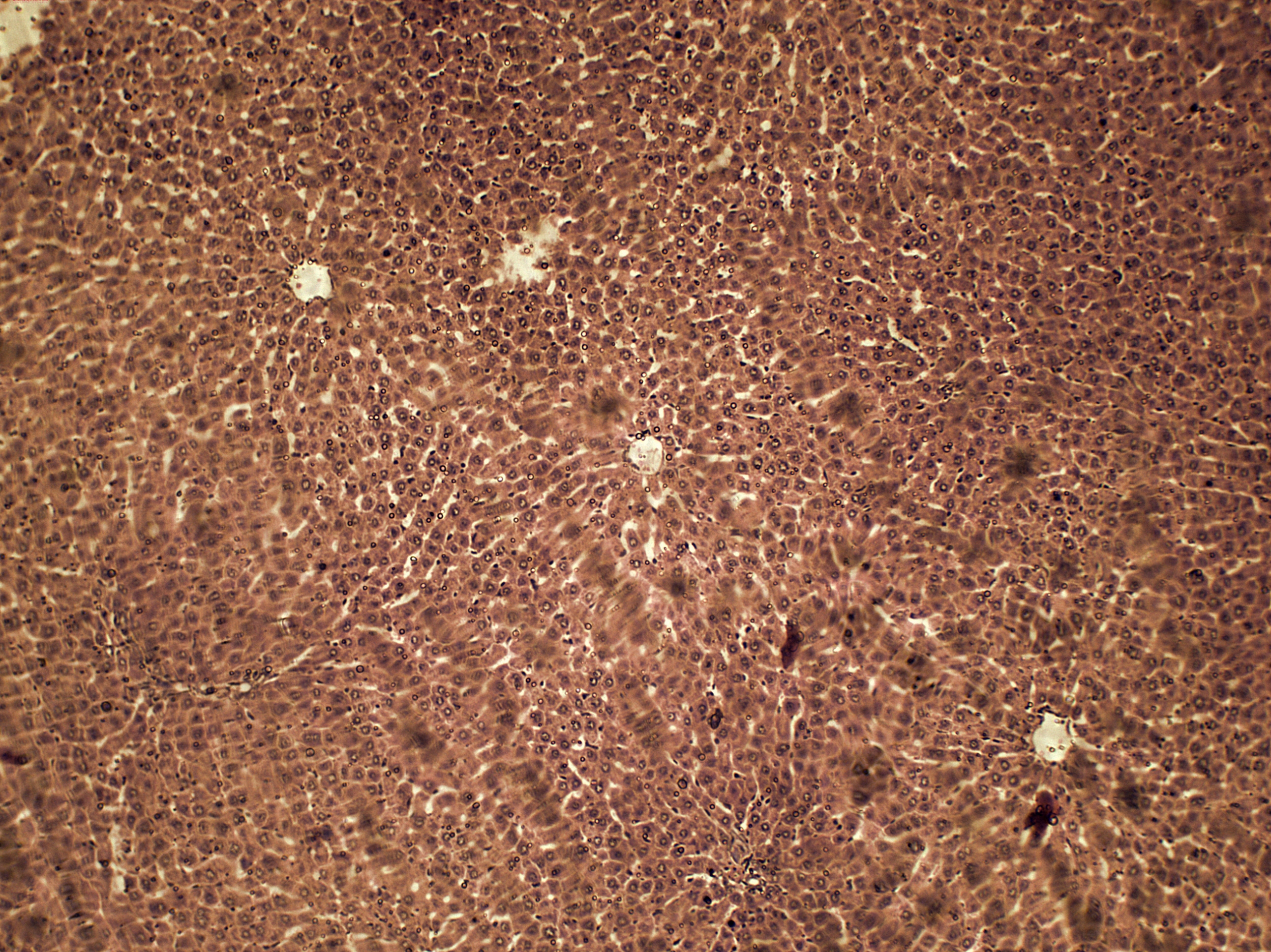

Supplement: Supplementary file 9 — Additional file 9. Fig Ad3f. Histopathological observations (10X) of Gp 6 liver. [file 12906_2020_2975_MOESM9_ESM.jpg]

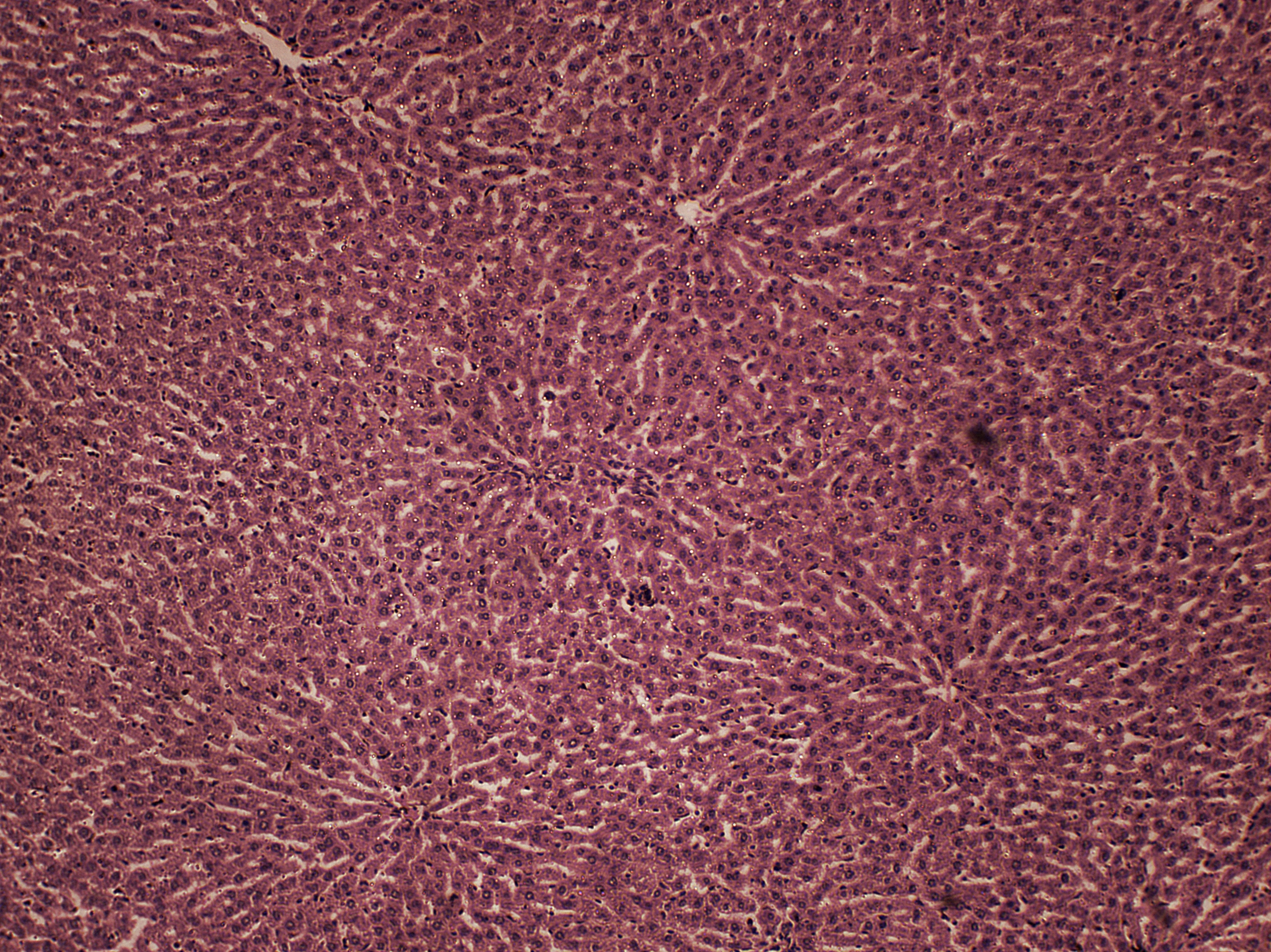

Supplement: Supplementary file 10 — Additional file 10. Fig Ad3g. Histopathological observations (10X) of Gp 7 liver. [file 12906_2020_2975_MOESM10_ESM.jpg]

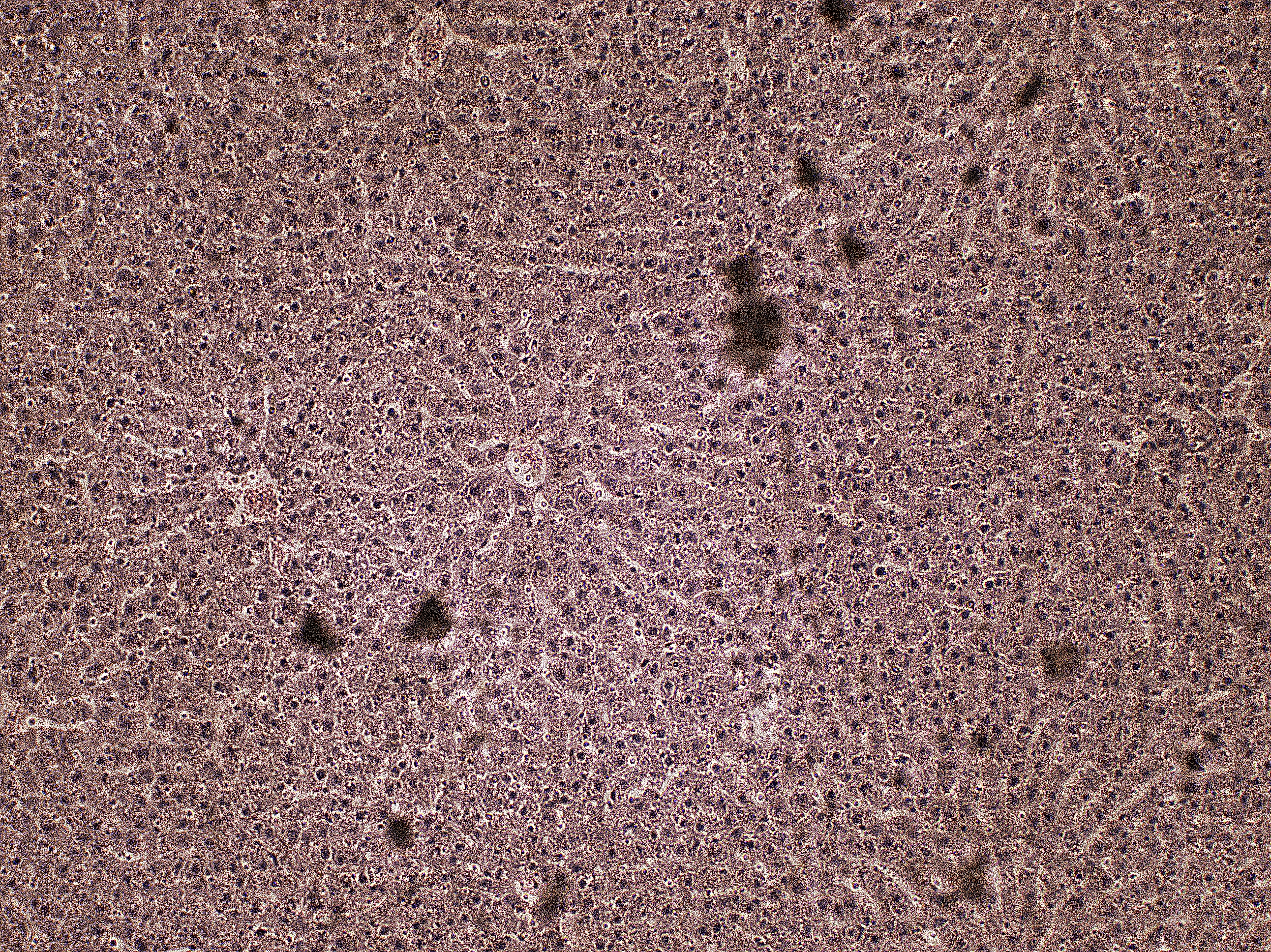

Supplement: Supplementary file 11 — Additional file 11. Fig Ad3h. Histopathological observations (10X) of Gp 8 liver. [file 12906_2020_2975_MOESM11_ESM.jpg]

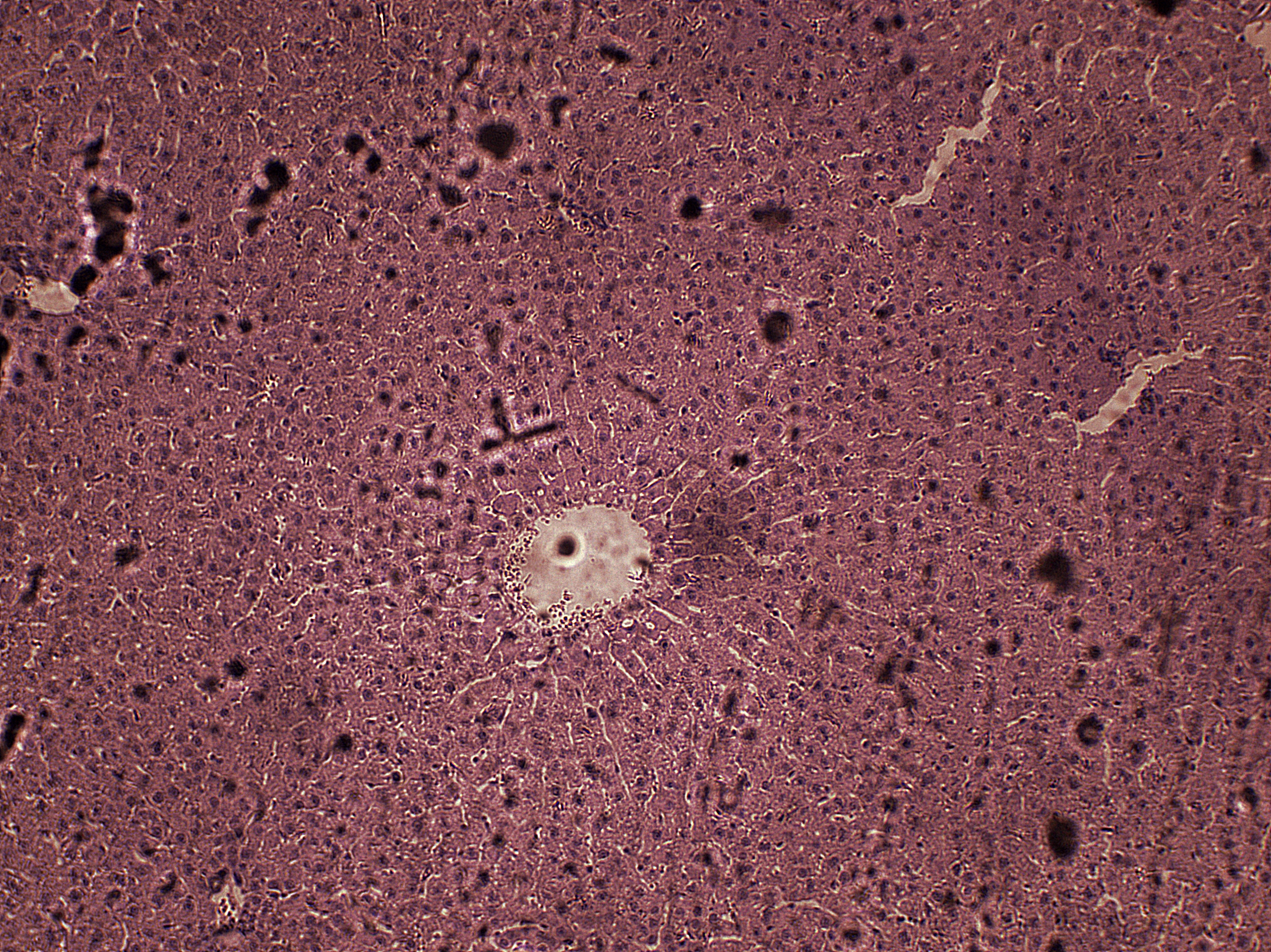

Supplement: Supplementary file 12 — Additional file 12. Fig Ad3i. Histopathological observations (10X) of Gp 9 liver. [file 12906_2020_2975_MOESM12_ESM.jpg]

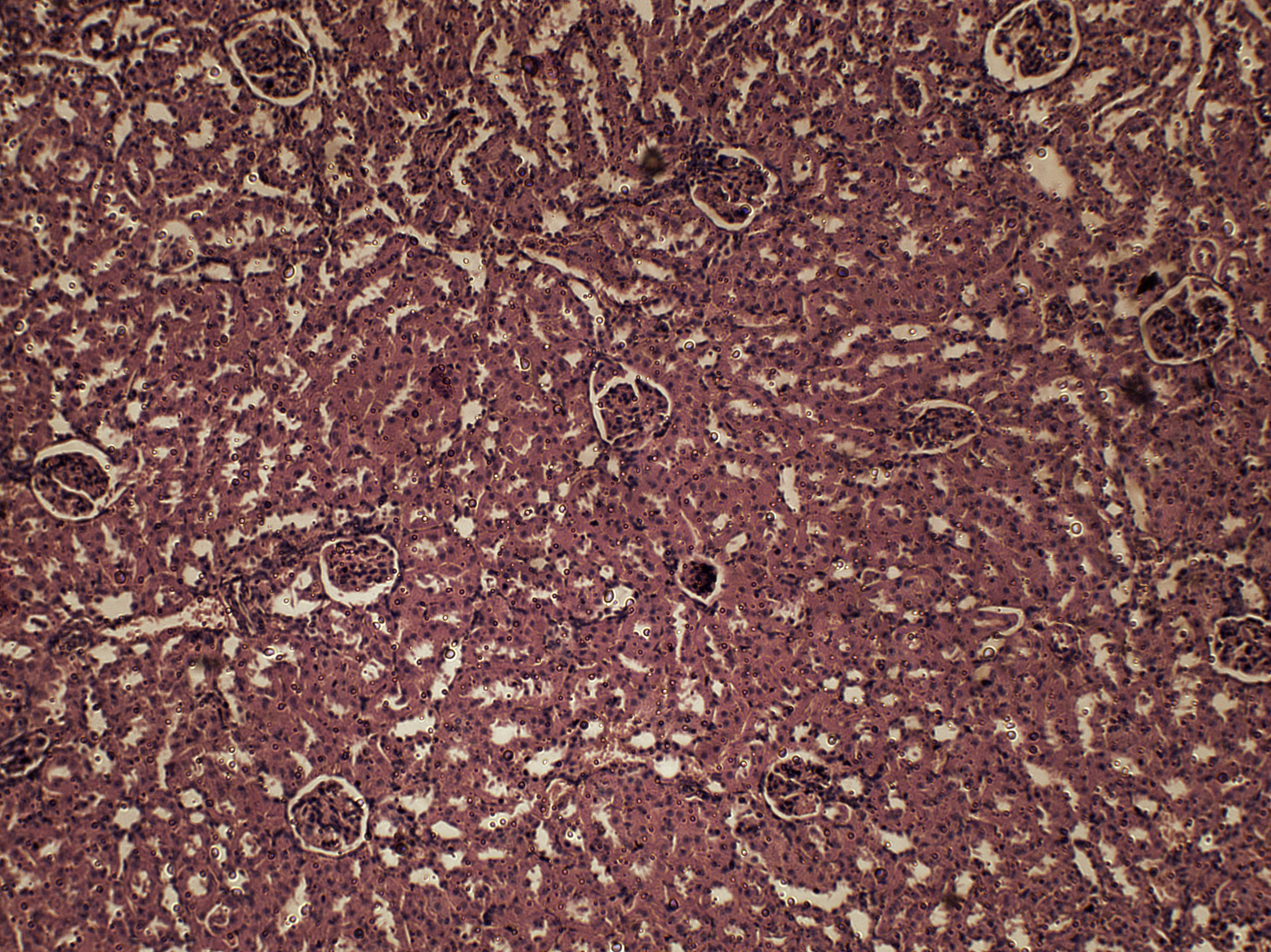

Supplement: Supplementary file 13 — Additional file 13. Fig Ad4a. Histopathological observations (10X) of Gp 1 Kidney. [file 12906_2020_2975_MOESM13_ESM.jpg]

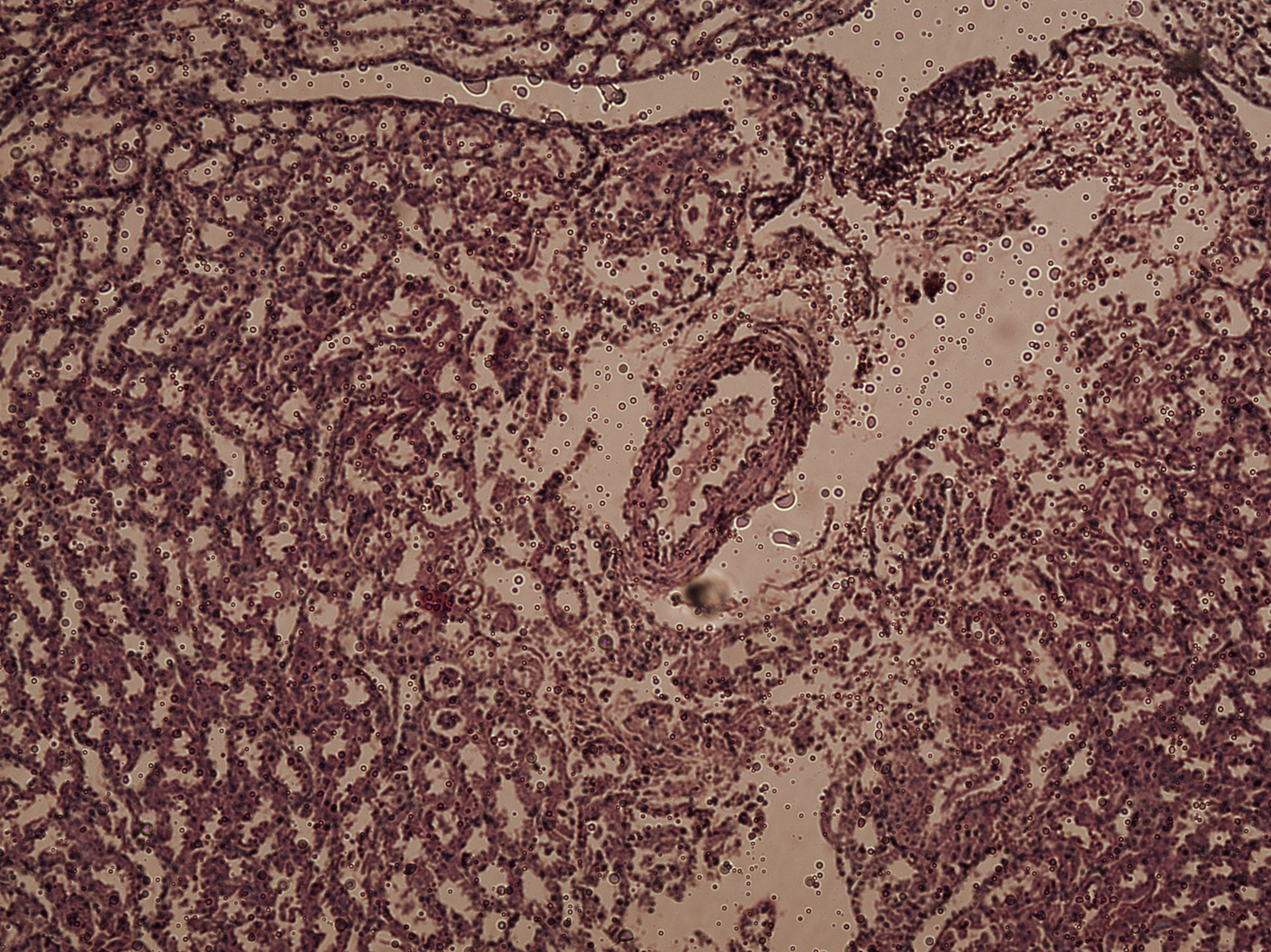

Supplement: Supplementary file 14 — Additional file 14. Fig Ad4b. Histopathological observations (10X) of Gp 2 Kidney. [file 12906_2020_2975_MOESM14_ESM.jpg]

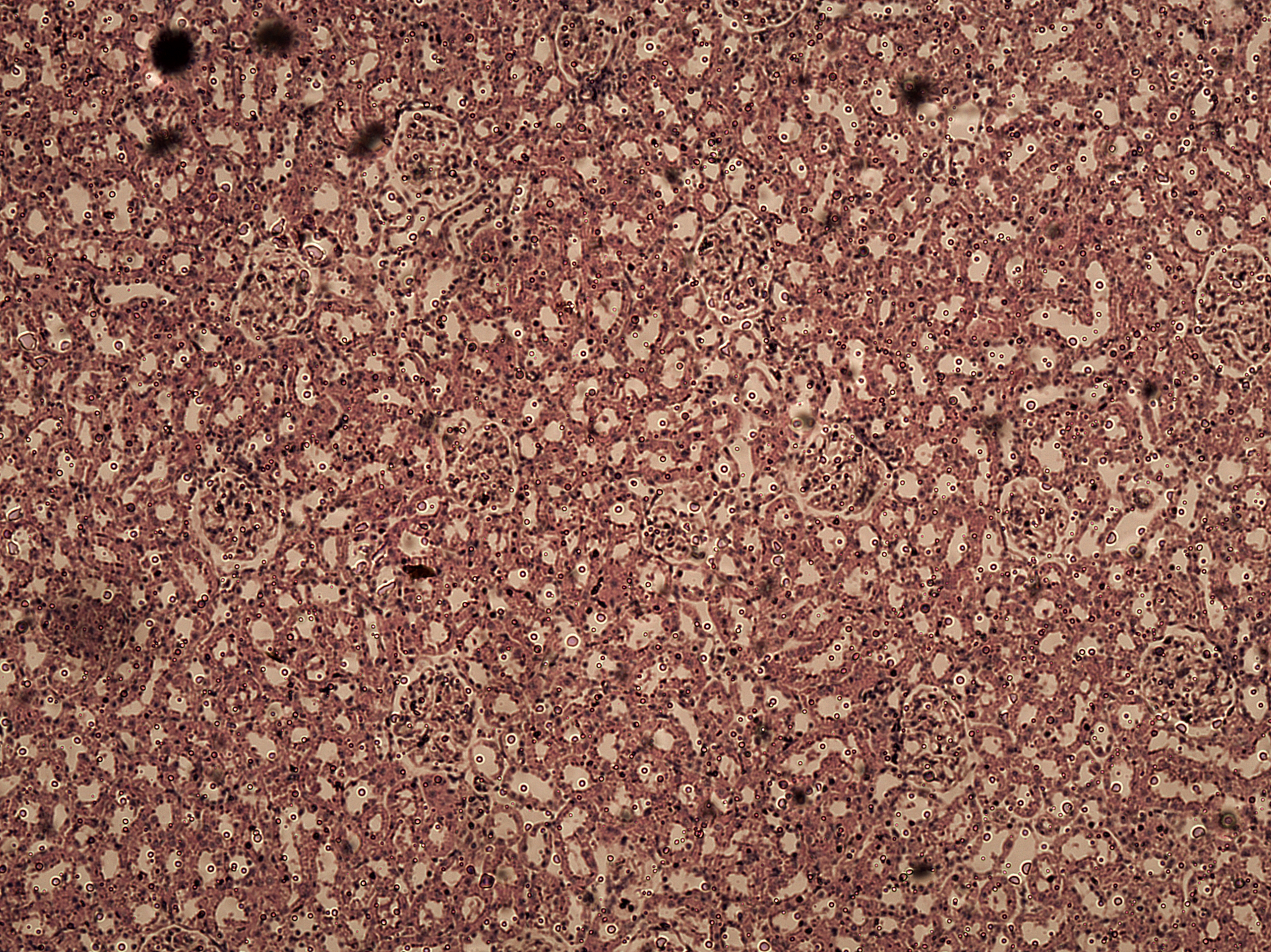

Supplement: Supplementary file 15 — Additional file 15. Fig Ad4c. Histopathological observations (10X) of Gp 3 Kidney. [file 12906_2020_2975_MOESM15_ESM.jpg]

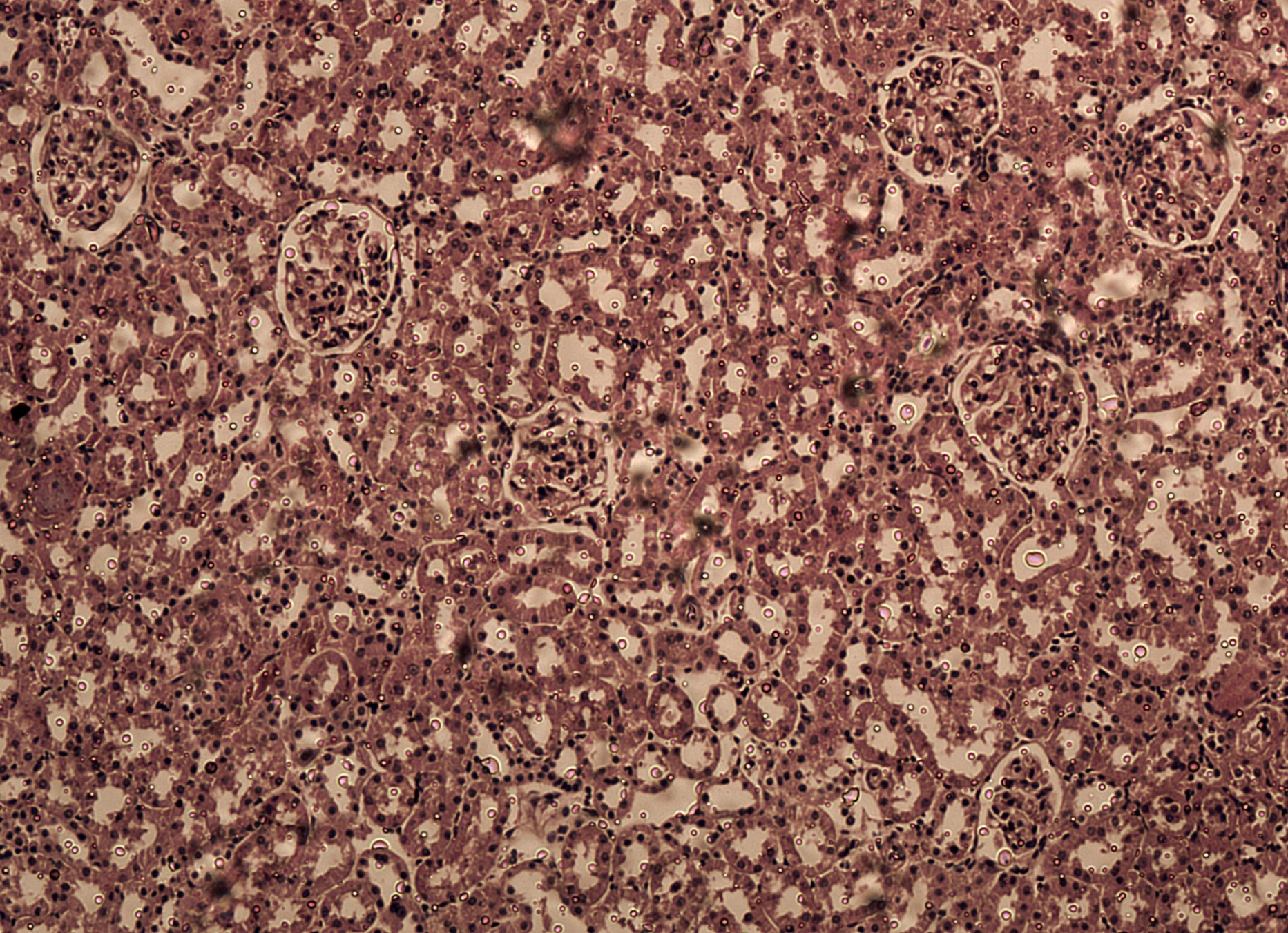

Supplement: Supplementary file 16 — Additional file 16. Fig Ad4d. Histopathological observations (10X) of Gp 4 Kidney. [file 12906_2020_2975_MOESM16_ESM.jpg]

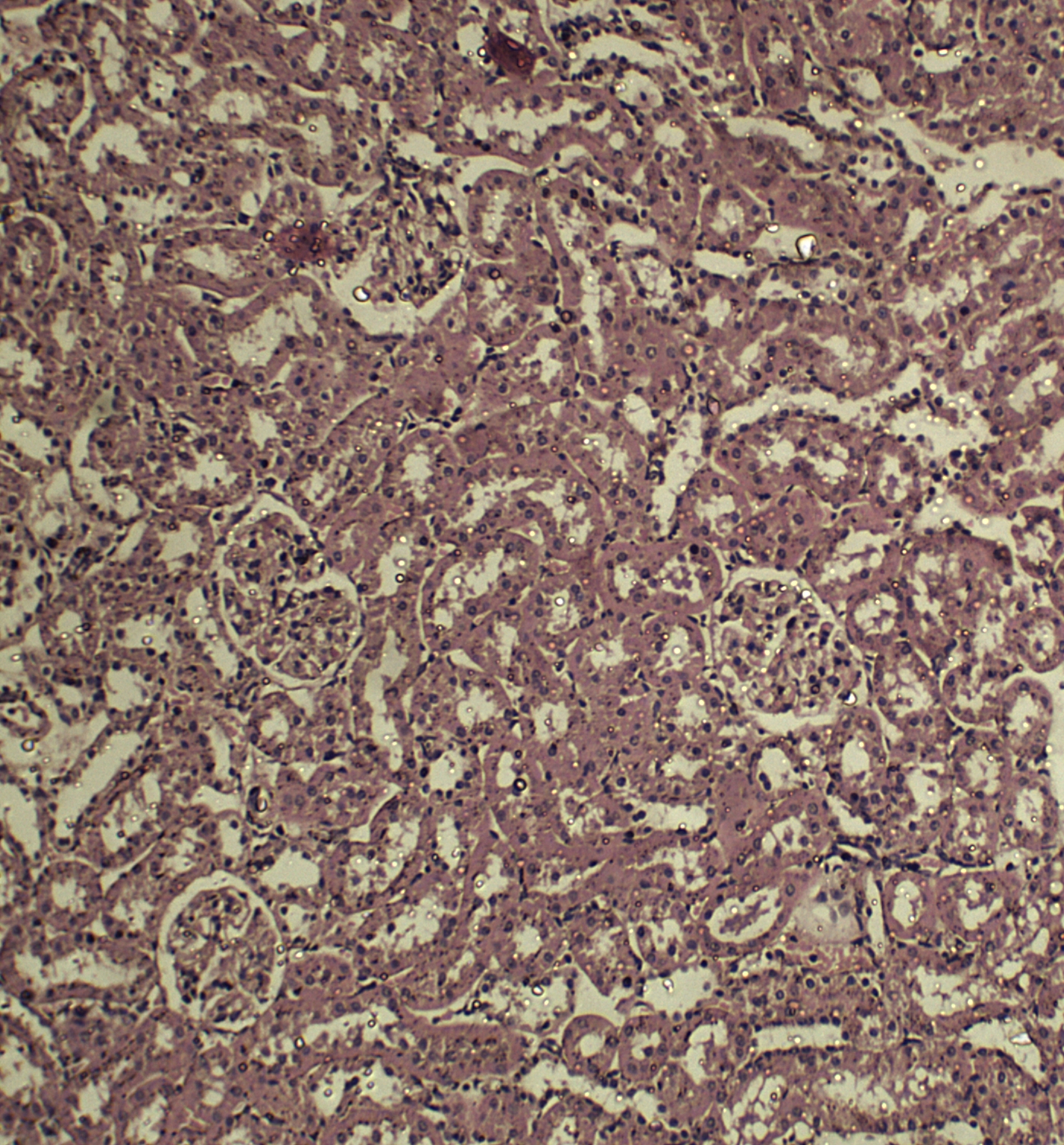

Supplement: Supplementary file 17 — Additional file 17. Fig Ad4e. Histopathological observations (10X) of Gp 5 Kidney. [file 12906_2020_2975_MOESM17_ESM.jpg]

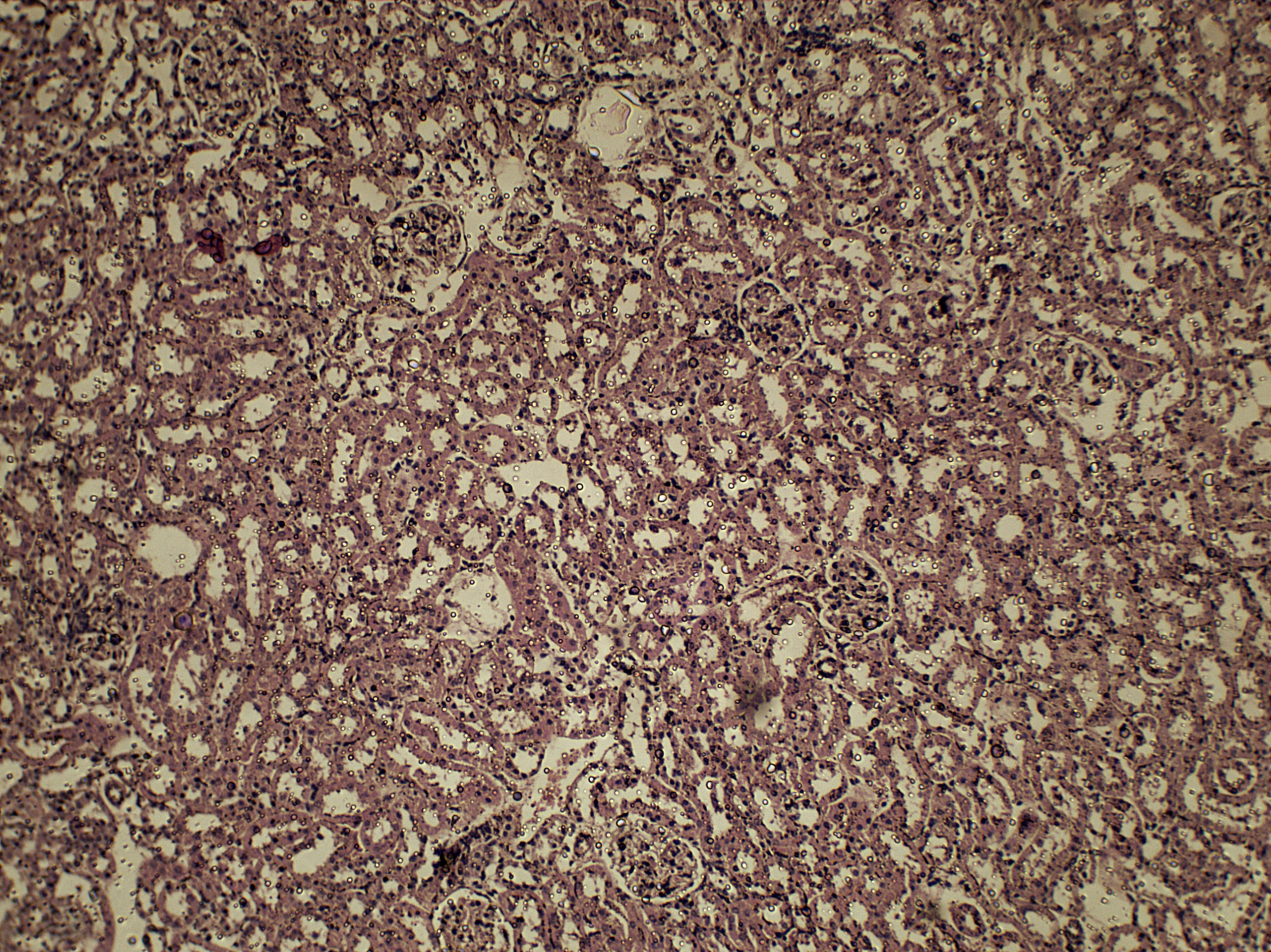

Supplement: Supplementary file 18 — Additional file 18. Fig Ad4f. Histopathological observations (10X) of Gp 6 Kidney. [file 12906_2020_2975_MOESM18_ESM.jpg]

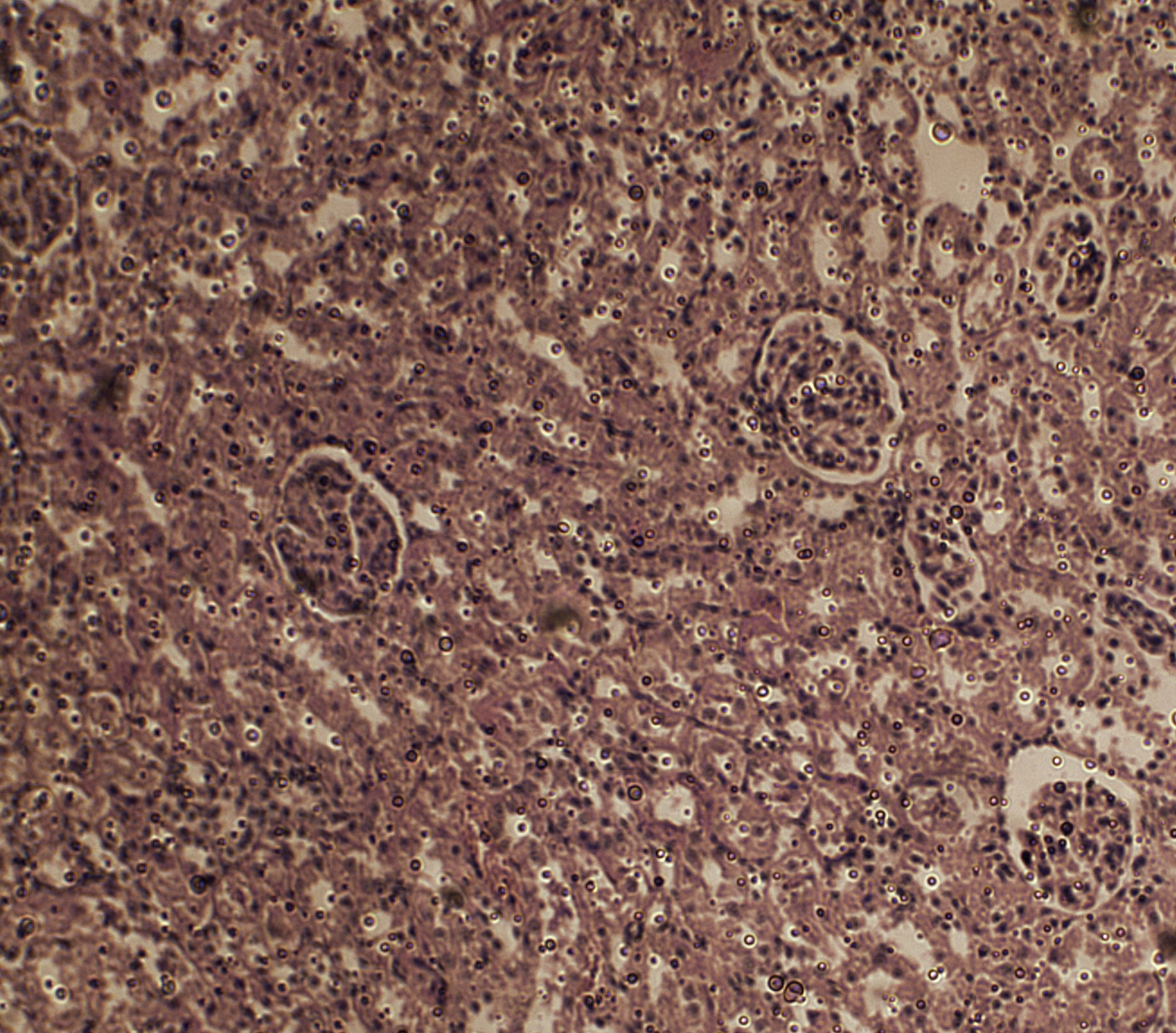

Supplement: Supplementary file 19 — Additional file 19. Fig Ad4g. Histopathological observations (10X) of Gp 7 Kidney. [file 12906_2020_2975_MOESM19_ESM.jpg]

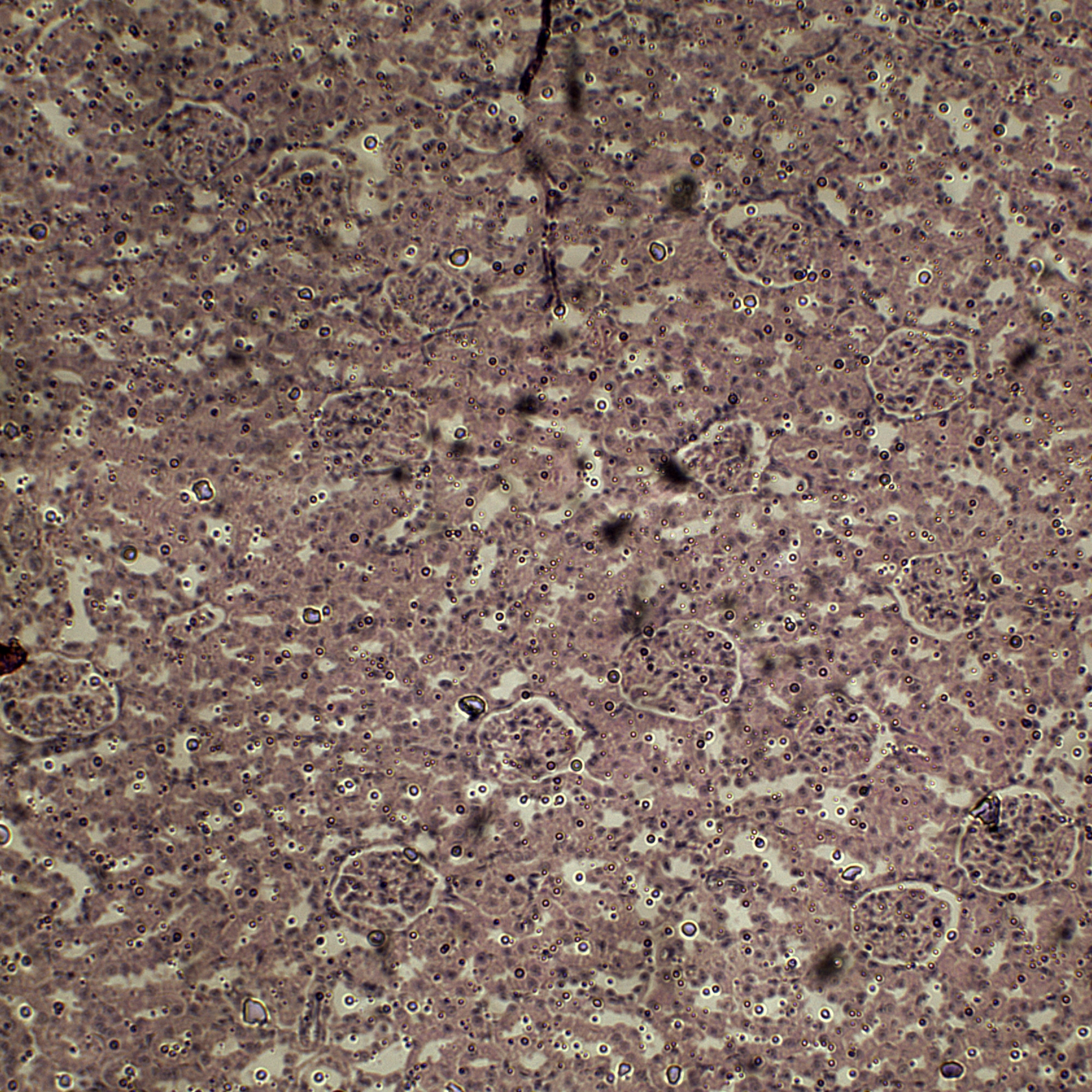

Supplement: Supplementary file 20 — Additional file 20. Fig Ad4h. Histopathological observations (10X) of Gp 8 Kidney. [file 12906_2020_2975_MOESM20_ESM.jpg]

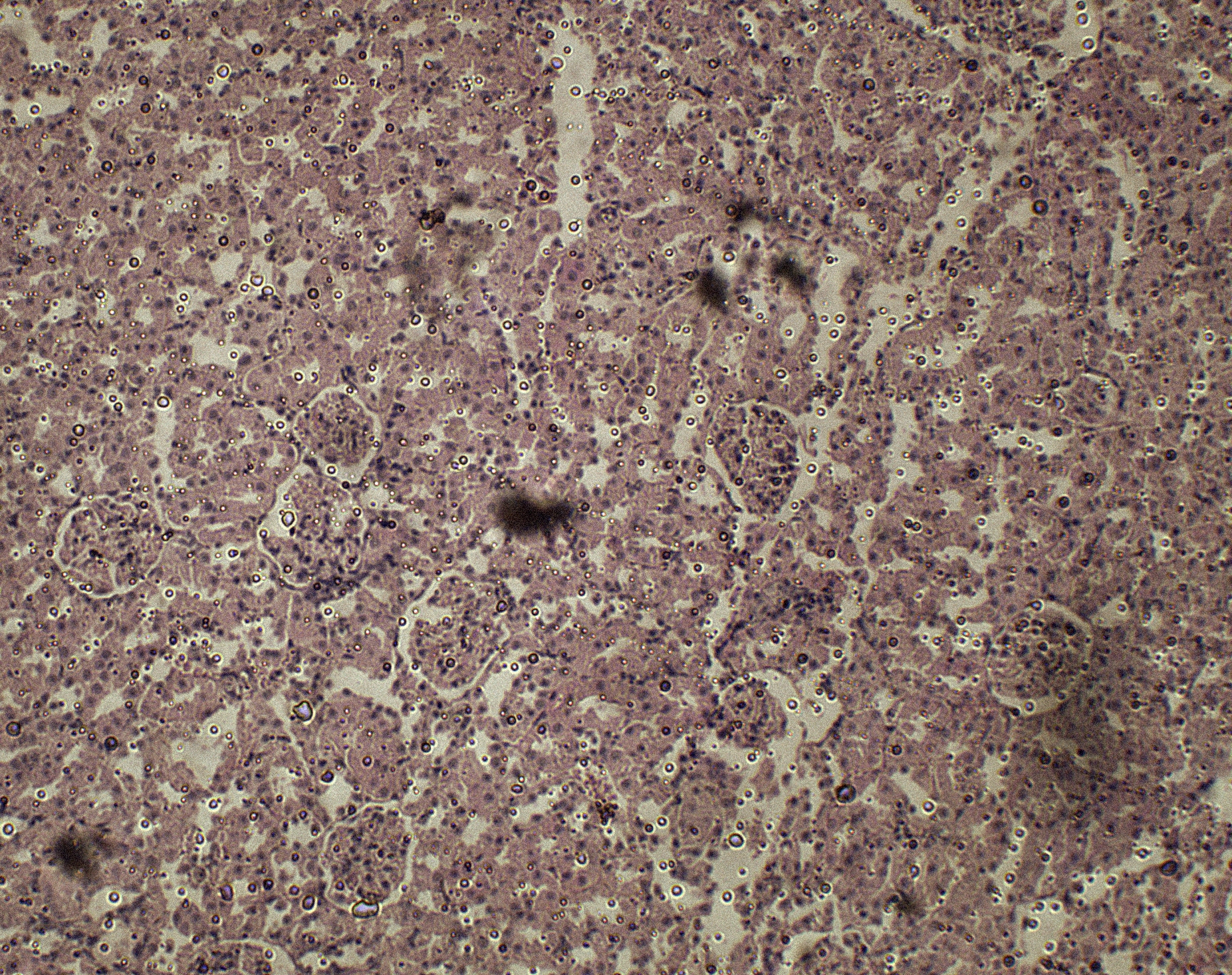

Supplement: Supplementary file 21 — Additional file 21. Fig Ad4i. Histopathological observations (10X) of Gp 9 Kidney. [file 12906_2020_2975_MOESM21_ESM.jpg]
